# Characterisation of a Japanese Encephalitis virus genotype 4 isolate from the 2022 Australian outbreak

**DOI:** 10.1038/s44298-024-00025-5

**Published:** 2024-05-10

**Authors:** Wilson Nguyen, Narayan Gyawali, Romal Stewart, Bing Tang, Abigail L. Cox, Kexin Yan, Thibaut Larcher, Cameron R. Bishop, Nicholas Wood, Gregor J. Devine, Andreas Suhrbier, Daniel J. Rawle

**Affiliations:** 1 QIMR Berghofer Medical Research Institute, Brisbane, QLD 4029 Australia; 2 INRAE, Oniris, PAnTher, APEX, Nantes, France; 3 National Centre for Immunisation Research and Surveillance, Westmead, NSW Australia; 4 GVN Center of Excellence, Australian Infectious Disease Research Centre, Brisbane, QLD 4029 and 4072 Australia

**Keywords:** Virology, Viral pathogenesis

## Abstract

Human infections with the Japanese encephalitis virus (JEV) are a leading cause of viral encephalitis. An unprecedented outbreak of JEV genotype 4 was recently reported in Australia, with an isolate (JEV_NSW/22_) obtained from a stillborn piglet brain. Herein we conduct a thorough characterization of JEV_NSW/22_ in three different mouse strains and in human cortical brain organoids (hBOs), and determined the ability of JEV_NSW/22_ to be neutralized by sera from humans vaccinated with IMOJEV. JEV_NSW/22_ was less virulent than JEV_FU_ (genotype 2) and JEV_Nakayama_ (genotype 3) in C57BL/6J mice and in interferon regulatory factor 7 deficient (*Irf7*^*−/−*^) mice, with infection of wild-type and knockout murine embryonic fibroblasts indicating JEV_NSW/22_ is more sensitive to type I interferon responses. *Irf7*^*−/−*^ mice provide a new model for JEV_NSW/22_, showing higher viremia levels compared to C57BL/6J mice, and allowing for lethal neuroinvasive infection. All JEV strains were universally lethal in *Ifnar*^*−/−*^ mice by day 3, with histological signs of brain hemorrhage, but no other lesions. There were no indications of brain infection in *Ifnar*^*−/−*^ mice, with viral protein detected in blood vessels, but not neurons. All JEV isolates showed robust cytopathic infection of human cortical brain organoids, albeit lower for JEV_NSW/22_. IMOJEV vaccination in humans induced antibodies capable of neutralizing JEV_NSW/22_, although, for all JEV strains, cross-neutralization titers declined with increasing divergence from IMOJEV in the envelope amino acid sequences. Overall, our study establishes JEV_NSW/22_ mouse and hBO models of infection, allowing for possible lethal neuroinvasive infection in mice that was rarer than for other JEV genotypes. JEV vaccination regimens may afford protection against this newly emerged JEV genotype 4 strain, although neutralizing antibody responses are sub-optimal.

## Introduction

Japanese encephalitis virus (JEV) is a single-stranded positive-sense RNA virus from the *Flaviviridae* family that is transmitted from amplifying hosts (primarily pigs and wading birds) via mosquitoes to humans^1^. While the infection is usually asymptomatic, encephalitis can develop in ≈1 in 250 people, with ~30% of encephalitic cases becoming fatal, and 30–50% of non-fatal encephalitic cases retaining persistent neurological symptoms including seizures, speech impediments, and paralysis^2,3^. JEV is the leading cause of viral encephalitis in Asia, with ~70,000 cases and ~20,000 deaths per annum^4^. After a bite from an infected mosquito, JEV replicates in peripheral blood monocytes producing a viremia that, in some cases, leads to the virus crossing the blood-brain barrier^5^. JEV primarily infects neurons in the brain^6^ leading to uncontrolled inflammation (encephalitis) and neuronal cell death^2^. There are several available and effective vaccines against JEV^7^, but there are no specific licensed treatments.

JEV exists as five genotypes (1–5) which are phylogenetically, antigenically, and geographically distinct^8–10^. Genotype 3 was the main genotype endemic in Asia until 1990, after which genotype I have dominated since^11^. Genotype 2 has been identified in Malaysia, Indonesia, Papua New Guinea and Australian outbreaks from 1970–2000. Genotype 5 was originally isolated in Malaysia in 1952, and has since been identified in China, and is now dominant in South Korea^12^. Genotype 4 was the least common genotype worldwide, having only been identified in mosquitoes from Indonesia and Papua New Guinea^13^. In February 2021, a fatal JEV infection occurred on the Tiwi Islands, 80 km off the coast of Darwin, Australia^14^. Sequencing identified that the virus belonged to the historically rare JEV genotype 4^14^. In 2022, a geographically widespread outbreak throughout most Australian states was attributed to JEV genotype 4, which caused 44 confirmed human cases and 7 deaths^15^. Outbreaks occurred in several piggeries, causing high abortion and stillbirth rates in sows^16^. Based on proximity to piggeries, estimates indicate that ~740,000 people may be at risk of being infected by JEV in Australia^16^. Given that *Culex annulirostris* mosquitoes, considered the primary vector for JEV in Australia^15,16^, and amplifying vertebrate hosts, including wading birds and pigs, are widespread, it is possible that JEV may become, or is already, endemic in Australia^15^. Murray Valley Encephalitis virus (MVEV) and Kunjin virus are phylogenetically closely related to JEV, have similar vector and reservoir hosts, and are endemic to Australia^17^.

Two JEV vaccines are licensed for use in Australia; IMOJEV (live attenuated) and JEspect (formalin inactivated). Before the 2022 outbreak, JEV vaccination primarily served as a travel vaccine for those traveling to Southeast Asian countries^18^. In response to the outbreak, a priority vaccination program was promptly instituted for people at higher risk of exposure. This included piggery workers, laboratory scientists handling JEV, mosquito surveillance scientists, and those residing in or frequently visiting high-risk locations^18^. Both IMOJEV and JEspect vaccines are designed against a JEV genotype 3 antigen, and there have been no studies assessing their efficacy against the genotype 4 strain responsible for the Australian outbreak.

Efforts to develop treatments for JEV neuropathology are hindered by the difficulties in diagnosing early infection^13^ before the virus has infected central nervous system (CNS) neurons; and once the virus has infected CNS neurons, treatments must also be able to effectively enter the brain. While a range of JEV mouse models have been reported (Supplementary Table 1)^19^, there are no studies of genotype 4 peripheral inoculation in adult mice, with the only studies on genotype 4 JEV using 3-week-old mice^20^ or intracranial inoculation^21^. Well-characterized, adult mouse models of recent genotype 4 isolates would represent useful tools to study mechanisms of infection and disease and evaluate potential new interventions. Herein, we compare and contrast a JEV genotype 4 isolate from the recent Australian outbreak (JEV_NSW/22_) with historical JEV isolates (genotypes 2 and 3) in three mouse strains, human cortical brain organoids and *in vitro* neutralization assays with human serum after JEV vaccination.

## Materials and methods

### Ethics statement and regulatory compliance

All mouse work was conducted in accordance with the “Australian code for the care and use of animals for scientific purposes” as defined by the National Health and Medical Research Council of Australia. Mouse work was approved by the QIMR Berghofer Medical Research Institute (MRI) Animal Ethics Committee (P3746, A2108-612). Mice were euthanized using CO_2_.

Breeding and use of GM mice were approved under a Notifiable Low Risk Dealing (NLRD) Identifier: NLRD_Suhrbier_Oct2020: NLRD 1.1(a). The use of IMOJEV was approved under NLRD_Suhrbier_Oct2019: NLRD2.1(d).

All infectious JEV work was conducted in a dedicated suite in a biosafety level-3 (PC3) facility at the QIMR Berghofer MRI (Australian Department of Agriculture, Water and the Environment certification Q2326 and Office of the Gene Technology Regulator certification 3445). All work was approved by the QIMR Berghofer MRI Safety Committee (P3746).

Human serum samples before and after IMOJEV vaccination were collected from 9 participants with human research ethics approval from Sydney Children’s Hospitals Network Human Research Ethics Committee (2022/ETH02471 HCHNHREC) for an ongoing project led by Dr. Nicholas Wood; “Japanese encephalitis vaccine via the intradermal route in children and adults (JEVID-2): Critical policy-relevant research for Australia”.

Human serum samples after IMOJEV vaccination were also collected from 10 participants with approval from QIMR Berghofer MRI Human Research Ethics Committee (P3476) provided for neutralization assays (including MVEV cross-neutralization).

Research with JEV was approved under the Queensland Biosecurity Act, Scientific Research Permit (restricted matter) – Permit number PRID000916.

### Cell lines and culture

Vero E6 (C1008, ECACC, Wiltshire, England; obtained via Sigma-Aldrich, St. Louis, MO, USA), BHK-21 (ATCC# CCL-10), and C6/36 cells (ATCC# CRL-1660) were cultured in medium comprising RPMI 1640 (Gibco), supplemented with 10% fetal bovine serum (FBS), penicillin (100 IU/ml)/streptomycin (100 μg/ml) (Gibco/Life Technologies) and L-glutamine (2 mM) (Life Technologies). Vero E6 and BHK cells were cultured at 37 °C and 5% CO_2_, and C6/36 cells were cultured at 27 °C and 5% CO_2_. Cells were routinely checked for mycoplasma (MycoAlert Mycoplasma Detection Kit MycoAlert, Lonza), and FBS was assayed for endotoxin contamination before purchase^22^.

Mouse embryonic fibroblasts (MEFs) were either wild-type or *Irf3/7*^*−/−*^ and have been described previously^23^. MEFs were cultured in Dulbecco’s Modified Eagle Medium (DMEM) (Gibco), supplemented with 50 μg/ml Penicillin/50 μg/ml Streptomycin and 10% Fetal Bovine Serum (FBS). Cells were cultured at 37 °C and 5% CO_2_. For virus growth kinetics, MEFs were seeded at 2 × 10^5^ cells/ml in 12 or 24 well plates one day prior to infection at multiplicity of infection (MOI) 0.1 of the indicated JEV or MVEV. After 1 hr incubation, MEFs were washed twice with 1 ml PBS, and culture medium was added and sampled daily for virus titrations by CCID_50_ (see below).

RENcell VM Human Neural Progenitor Cell Line (Sigma-Aldrich) were cultured in medium comprising DMEM F-12 (Thermo Fisher Scientific), penicillin (100 IU/ml)/streptomycin (100 μg/ml) (Gibco/Life Technologies), 20 ng/ml FGF (STEMCELL Technologies), 20 ng/ml EGF (STEMCELL Technologies), and 2% B27 supplements (Thermo Fisher Scientific). Cells were detached using StemPro Accutase Cell Dissociation Reagent (Thermo Fisher Scientific), and were cultured in Matrigel (Sigma). For crystal violet staining of remaining cells after infection, formaldehyde (7.5% w/v)/crystal violet (0.05% w/v) was added to wells overnight, plates washed twice in water, and plates dried overnight.

### Virus isolates and culture

JEV_Nakayama_ (GenBank: EF571853), JEV_FU_ (GenBank: AF217620), and IMOJEV were obtained from A/Prof. Gregor Devine (QIMR Berghofer MRI). JEV_NSW/22_ (GenBank: OP904182) was obtained from an infected porcine neonate by Dr. Peter Kirkland (Elizabeth Macarthur Agriculture Institute, New South Wales, Australia). MVEV_TC123130_ (GenBank: JN119814.1) was obtained from the “Doherty Virus Collection” currently held at QIMR Berghofer MRI. Virus stocks were generated by infection of C6/36 cells (for JEV and MVEV) or Vero E6 cells (for IMOJEV and YFV 17D) at MOI ≈ 0.1, with supernatant collected after ~5 days, cell debris removed by centrifugation at 3000 × *g* for 15 min at 4 °C, and virus aliquoted and stored at −80 °C. Virus stocks used in these experiments underwent less than three passages in our laboratory, with prior passage history in Supplementary Fig. 1. Virus titers were determined using standard CCID_50_ assays (see below).

#### Validation of virus stock sequences

Viral RNA was extracted from virus stock culture supernatants using NucleoSpin RNA Virus kit (Machery Nagel) as per manufacturer’s instructions. cDNA was synthesized using ProtoScipt First Strand cDNA Synthesis Kit (New England Biolabs) as per manufacturer’s instructions. PCR was performed using Q5 High-Fidelity 2X Master Mix (New England Biolabs) as per manufacturer’s instructions with the following primers; JEV envelope Forward 5’ GGAAGCATTGACACATGTGC 3’ and Reverse 5’ TCTGTGCACATACCATAGGTTGTG 3’, and MVEV envelope Forward 5’ GAGCATTGACACATGCGCAAAG and Reverse 5’ TGTGCACATCCCATAAGTGGTTC 3’. PCR products were run on a 1% agarose gel, and DNA was extracted using Monarch DNA Gel Extraction Kit (New England Biolabs). DNA was sequenced by Sanger sequencing using either the forward or reverse primer. Sequences of our virus stocks matched the sequences on GenBank (Supplementary Fig. 1).

### Cell culture infectious dose 50% (CCID_50_) assays

CCID_50_ assays were performed as previously described^24–26^. C6/36 cells were plated in 96 well flat bottom plates at 2 × 10^4^ cells per well in 100 µl of medium. For tissue titrations, tissues were homogenized in tubes each containing 4 ceramic beads twice at 6000 × *g* for 15 seconds, followed by centrifugation twice at 21,000 × *g* for 5 min. Samples underwent 10-fold serial dilutions in 100 µl RPMI 1640 supplemented with 2% FBS, performed in quadruplicate for tissue homogenate and in duplicate for serum and cell culture supernatant. For mouse tissues and serum, a volume of 100 µl of serially diluted samples was added to each well of 96 well plates containing C6/36 cells, and the plates were cultured for 5 days at 37 °C and 5% CO_2_. 25 µl of supernatant from infected C6/36 cells were then passaged on to Vero E6 cells plated the day before at 2 × 10^4^ cells per well in 96 well flat bottom plates. Supernatants from in vitro cultures were titered directly onto Vero E6 cells. Vero E6 cells were cultured for 5 days cytopathic effect was scored, and the virus titer was calculated using the method of Spearman and Karber^27^ (a convenient Excel CCID50 calculator is available at https://www.klinikum.uni-heidelberg.de/zentrum-fuer-infektiologie/molecular-virology/welcome/downloads).

### Mouse infections

C57BL/6J mice were purchased from the Animal Resources Center, Canning Vale WA, Australia. *Ifnar*^*−/−*^ were kindly provided by Dr P. Hertzog (Monash University, Melbourne, Australia). *Irf7*^*−/−*^ mice were kindly provided by T. Taniguchi (University of Tokyo)^28–30^. C57BL/6N mice were purchased from The Jackson Laboratory (stock no. 005304). C57BL/6NJ^Δ*Nnt8-12*^ were generated by The Australian Phenomics Network, Monash University, Melbourne, Australia, as described^31^. Mice used in this study were female, except *Irf7*^*−/−*^ mice where both males and females were used. The age/age range at infection is indicated in the figure legends. Mice were sorted into groups so that each group had a similar mean age and age distribution, and in the case of *Irf7*^*−/−*^ mice, had equal numbers of males and females in each group. All mice strains except C57BL/6J were bred in-house at QIMR Berghofer MRI, and mice housing conditions were described previously^32^. Mice were infected subcutaneously (s.c.) at the base of the tail with 100 µl of virus inoculum (doses ranging from 5 × 10^2^ to 5 × 10^5^ as indicated in the figure legends). Serum was collected via tail nicks into Microvette Serum-Gel 500 µl blood collection tubes (Sarstedt, Numbrecht, Germany). Mice were weighed and monitored for disease manifestations and were humanely euthanized using CO_2_ based on a scorecard system (Supplementary Fig. 2B). At necropsy, brain, and spleens were collected for virus titrations by CCID_50_ assays and/or for histology.

### Histopathology and immunohistochemistry

Brains and spleens were fixed in 10% formalin and embedded in paraffin. Human cortical brain organoids were embedded in agarose by adding 4% melted agarose and incubating on ice to solidify, prior to standard paraffin embedding. Sections were stained with H&E (Sigma-Aldrich), and slides were scanned using Aperio AT Turbo (Aperio, Vista, CA USA) and analyzed using Aperio ImageScope software (LeicaBiosystems, Mt Waverley, Australia) (v10). Leukocyte infiltrates were quantified by measuring nuclear (strong purple staining)/cytoplasmic (total red staining) pixel ratios in scanned H&E stained images, and was undertaken using Aperio Positive Pixel Count Algorithm (Leica Biosystems)^33^.

For anti-flavivirus non-structural protein 1 (NS1) immunohistochemistry using 4G4, sections were affixed to positively charged adhesive slides and air-dried overnight at 37 °C. Sections were dewaxed and rehydrated through xylol and descending graded alcohols to water. Sections were transferred to Dako Epitope Retrieval Solution (pH 9.0) and subjected to heat antigen retrieval (100 °C for 20 min) using the Biocare Medical de-cloaking chamber, and slides allowed to cool for 20 minutes before transferring to TBS plus 0.025% Tween-20 (TBS-T). Endogenous mouse Ig was blocked by incubating sections with donkey anti-mouse Fab fragments (Jackson Immunoresearch) diluted 1:50 in Biocare Medical Rodent block M for 60 minutes. Sections were washed three times in TBS-T, then incubated with anti-mouse Fc for 15 minutes, before a further three washes in TBS-T. Nonspecific antibody binding was inhibited by incubation with Biocare Medical Background Sniper with 1% BSA 20% donkey serum, and 20% goat serum for 15 minutes. Primary antibody 4G4 (mouse anti-flavivirus NS1^25,34,35^) was diluted 1 in 4 with Da Vinci Green diluent and applied to the sections overnight at room temperature. Sections were washed three times in TBS-T, and endogenous peroxidase was blocked by incubating slides in Biocare Medical Peroxidased 1 for 5 minutes. Sections were washed three times in TBS-T, and Perkin Elmer Opal HRP Polymer or Perkin Elmer Goat anti-mouse HRP diluted 1:500 in TBS-T was applied for 60 minutes. Sections were washed three times in TBS-T, and signal developed in Vector Nova Red for 5 minutes, after which they were washed three times in water. Sections were lightly counterstained in Haematoxylin (program 7 Leica Autostainer), washed in water, dehydrated through ascending graded alcohols, cleared in xylene, and mounted using DePeX or similar.

Apoptosis was detected using the ApopTag Peroxidase In Situ Apoptosis Detection Kit (Merck Catalog No. S7100) as per the manufacturer’s instructions.

For anti-GFAP IHC, antigen retrieval was performed in 0.1 M citric acid buffer (pH 6.0) at 1005 °C for 20 min. Endogenous peroxidase activity was blocked using 1.0% H_2_O_2_ and 0.1% sodium azide in TBS for 10 min. Endogenous mouse Ig was blocked by incubating sections with goat anti-mouse Fab fragments (Jackson Immunoresearch) diluted 1:50 in Biocare Medical Mouse block M for 60 minutes. Nonspecific antibody binding was inhibited by incubation with Biocare Medical Background Sniper + 2% BSA for 30 min. Mouse anti-GFAP clone GA-5 (Biocare Medical, CM065C), was diluted 1:250 in the above buffer and incubated in sections for 1 hr at room temperature. After washes, Perkin Elmer Goat anti-mouse HRP diluted 1:500 in TBS-T was applied for 60 minutes. Nova Red development and counter staining was performed as for 4G4 above.

### Infection of human cortical brain organoids

hBOs were reprogrammed from adult dermal fibroblasts (HDFa, Gibco, C0135C) using the CytoTune-iPS 2.0 Sendai Reprogramming Kit (Invitrogen, A16518)^36^, and were grown using the CelVivo Clinostar incubator (Invitro Technologies) as described^37^. On the day of infection, ~30-day-old hBOs were transferred from each Clinoreactor into an ultra-low-binding 24-well plate (one hBO per well), and each hBO was infected with 10^5^ CCID_50_ of either JEV_Nakayama_, JEV_FU_, JEV_NSW/22_, MVEV_TC123130_, IMOJEV^38^, or YFV 17D^25^ for ~4 h. For shorter-term culture (up to 4 days for viral growth kinetics), virus inoculum was removed, and hBOs were washed twice with media in the well, before 1 ml differentiation media was added to each well, and the 24-well plate was placed within a humidified tissue culture incubator at 37 °C, 5% CO_2_ for up to 4 days. For culture up to 11 dpi, hBOs were washed twice with media by transferring them to an ultra-low-binding six-well plate containing 5 ml of media, and then hBOs were transferred into 50 mm LUMOX gas exchange dishes (SARSTEDT) (4 organoids per dish) containing 7 ml of differentiation media, and placed within a humidified tissue culture incubator at 37 °C, 5% CO_2_ for up to 11 days. hBOs were imaged using an EVOS FL (Advanced Microscopy Group), and organoid 2D image circumference was determined by drawing around the edge of the organoid using Image J v1.53^39^.

### Human serum neutralization assays

Cohort 1 is human serum samples from 9 participants (age >50 years), and were collected ~28 days (±2 days) after vaccination (administered via either subcutaneous or intradermal injection) with the IMOJEV vaccine (Sanofi-Aventis Australia). Serum was also collected pre-IMOJEV vaccination for these same participants for baseline analysis. Cohort 2 is human serum samples from 10 participants (age range 24 to 60 years), collected at variable times post-vaccination (~ 2 months to 1 year). Pre-vaccination serum was not collected for Cohort 2, and the vaccine was administered subcutaneously. Neutralizing antibodies against IMOJEV, JEV_Nakayama_, JEV_FU_, JEV_NSW/22_, and MVEV_TC123130_ were measured by plaque reduction neutralization (PRNT) assay^40,41^. BHK-21 cells were seeded at 1.6 × 10^5^ cells per well in 24 well plates overnight at 37 °C. Serum samples were heat-inactivated (56 °C, 30 min) and serially diluted fourfold from 1:5 to 1:160 in BHK-21 cell culture media. The serum was then incubated with 100–110 pfu of IMOJEV, JEV_Nakayama_, JEV_FU_, JEV_NSW/22_, and MVEV_TC123130_ for 1 h at 37 °C. Serum plus virus mixtures were then added to BHK-21 cell monolayers and incubated for 1 hr at 37 °C to enable non-neutralized virus to adsorb to cells. Thereafter, 1 ml of 0.375% w/v carboxymethyl cellulose (CMC, Sigma-Aldrich)/RPMI 1640 was added, and the plates were incubated at 37 °C in a CO_2_ incubator for 3 days for JEV_Nakayama_ and 4 days for JEV_NSW/22_. The CMC medium was then removed, and the cell monolayers were fixed and stained with 0.1% w/v crystal violet (Sigma-Aldrich) in formaldehyde (1% v/v) and methanol (1% v/v). Plate wells were washed with tap water, dried and the plaques were counted. The PRNT_50_ titer was interpolated from plaque count compared to the average plaque count for the naive or no serum control.

### Envelope protein structure visualizations

JEV envelope protein structure was downloaded from the Protein Data Bank (PDB: 5WSN)^42^. Envelope structure visualizations were generated using PyMol Molecular Graphics System (version 2.3.3; Schrodinger, NY, USA). Virus sequences were downloaded from GenBank (see Supplementary Fig. 1 for accession numbers) and aligned using Mega-X (Molecular Evolutionary Genetics Analysis 10, Penn State University, State College, PA, USA) and the ClustalW plugin with default parameters. Differences in amino acids compared to IMOJEV were colored according to structural conservation as described^43^.

### Statistics

The *t* test was used when the difference in variances was <4-fold, skewness was >−2, and kurtosis was <2 (Excel 2016). Otherwise, the non-parametric Kolmogorov–Smirnov exact test or Mann-Whitney test was used (GraphPad Prism 8). Paired *t* test was used for comparing neutralization of different viruses with the same human serum sample. For matched human serum samples, a paired non-parametric Wilcoxon matched-pairs signed rank test (GraphPad Prism 8) was used since the difference in variance was >4-fold. Kaplan–Meier statistics were determined by log-rank (Mantel–Cox) test. Area under the curve analyses was performed in GraphPad Prism 8, with an area under the curve values then compared by *t* test. Correlation analyses of PRNT_50_ with envelope conservation used the non-parametric Spearman’s rank-order correlation.

## Results

### JEV_NSW/22_ infection produces a viremia but is not lethal in C57BL/6J mice

The JEV_NSW/22_ G4 virus was isolated from the brain of a stillborn piglet in New South Wales (NSW), Australia, in February 2022 (GenBank accession OP904182). The JEV_Nakayama_ genotype 3 prototype was isolated in Japan in 1935 (GenBank accession EF571853), and the JEV_FU_ genotype 2 was isolated in Australia in 1995^44,45^ (GenBank accession AF217620). MVEV was isolated in Australia in 1974^46^ (MVEV_TC123130_, GenBank accession JN119814). The latter three viruses were isolated from human patients. Brief descriptions of the viral isolates and their phylogenic relationships with other flaviviruses are provided in Supplementary Fig. 1.

**Fig. 1:**
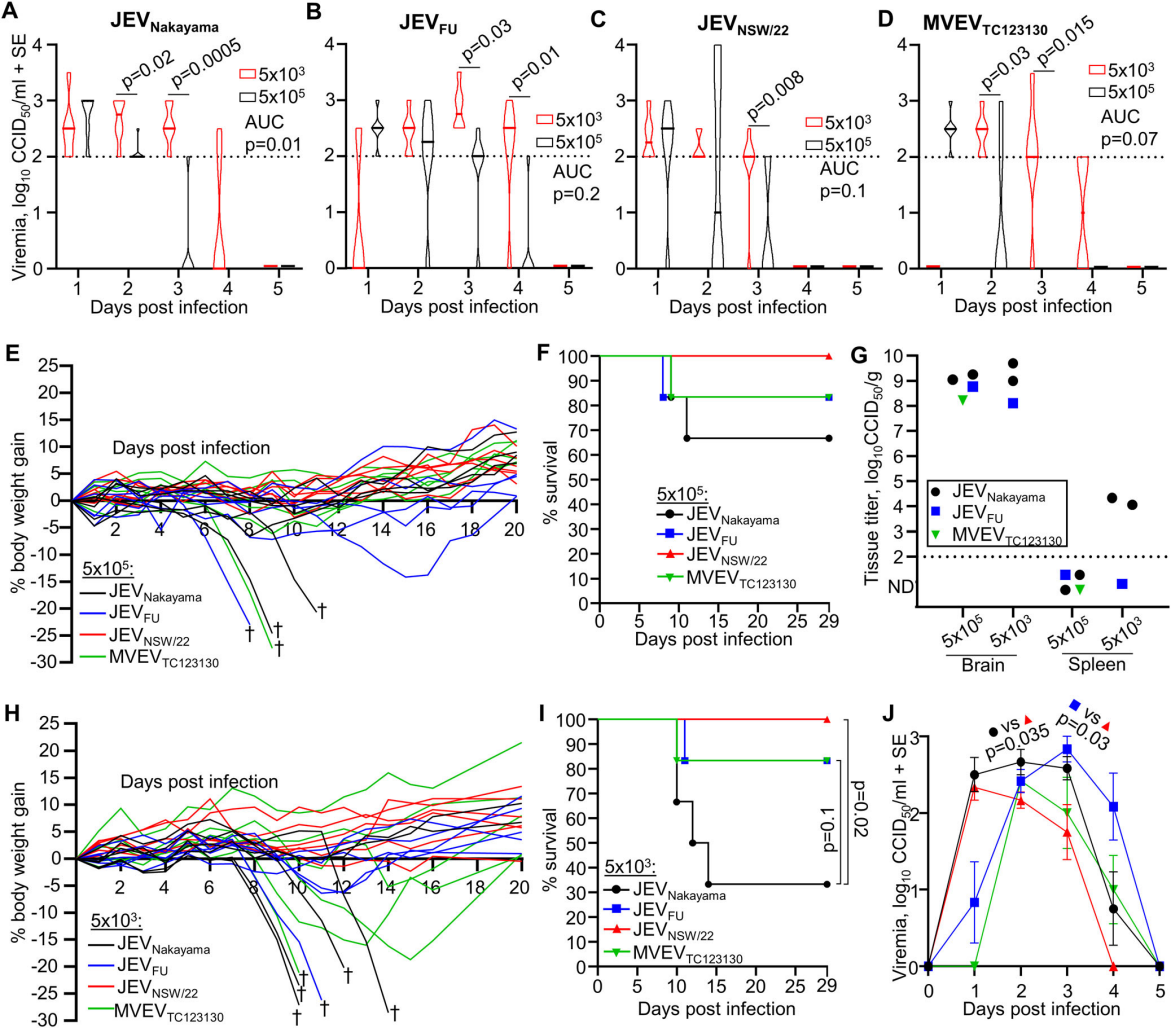
JEV and MVEV infection in C57BL/6J mice. **A**–**D** Female ≈6 week old C57BL/6J mice were infected s.c. with 5 × 10^5^ (black) or 5 × 10^3^ (red) CCID_50_ of the indicated viruses. Violin plots for *n* = 6 per group over 5 days is shown. The horizontal line within the violin plot represents the median. All mice recorded a detectable viremia for at least one timepoint. Limit of detection is 2 log_10_CCID_50_/ml of serum. Statistics represent *t* test or Kolmogorov–Smirnov exact test at the indicated timepoint or for area under the curve (AUC) values (see methods section). **E** Percent body weight change of individual mice after infection with the indicated virus at 5 × 10^5^ CCID_50_ compared to each mouse’s weight on day zero. Four mice lost ≥20% body weight and were euthanized (†). **F** Kaplan–Meier plot showing percent survival (*n* = 6 for each virus/virus isolate inoculated at 5 × 10^5^ CCID_50_). **G** Viral tissue titers in brains and spleens of seven euthanized mice at the time when the criteria for humane euthanasia was met (see Supplementary Fig. 2) (*n* = 4 JEV_Nakayama_—black circles, *n* = 2 JEV_FU_—blue square, *n* = 1 MVEV_TC123130_ green downward triangle). Tissue titers determined by CCID_50_ assay (limit of detection ~2 log_10_CCID_50_/g). **H** Percent body weight change of individual mice after infection with the indicated virus at 5 × 10^3^ CCID_50_ compared to each mouse’s weight on day zero. Six mice lost ≥20% body weight and were euthanized (†). **I** Kaplan-Meier plot showing percent survival (*n* = 6 for each virus/virus isolate inoculated at 5 × 10^3^ CCID_50_). **J** Viremia comparing JEV_Nakayama_ (black circles), JEV_FU_ (blue squares)_,_ JEV_NSW/22_ (red triangles) and MVEV_TC123130_ (green downward triangles) at 5 × 10^3^ inoculation dose; data is a reanalysis of data presented in Fig. 1A–D. Data are mean of *n* = 6 per group and error bars represent standard error. Statistics are *t* test for JEV_Nakayama_ versus JEV_NSW/22_ on day 2 and JEV_FU_ versus JEV_NSW/22_ on day 3.

Six-week-old adult C57BL/6J mice were infected subcutaneously (s.c.) with 5 × 10^5^ CCID_50_ of the aforementioned viruses. Viremia for all four viruses was broadly similar, peaking 1 day post infection (dpi) at 2–3 log_10_CCID_50_/ml of serum, with nearly all mice showing no detectable viremia by day 4 (Fig. 1A–D). The infection appeared to stall weight gain for most mice until day ~10 (Fig. 1E). Between 8 and 12 dpi, four mice (2 infected with JEV_Nakayama_, 1 JEV_FU_ and 1 MVEV_TC123130_) out of the total of 24 mice showed weight loss ≥20% and were euthanized (Fig. 1E, †). A further four mice (1 JEV_Nakayama_, 2 JEV_FU_, 1 MVEV_TC123130_) lost >5% of their body weight, but subsequently recovered. None of the JEV_NSW/22_ infected mice lost more than ~3% of their body weight (Fig. 1E). Kaplan-Meier survival curves provided no significant differences for the different viral isolates (Fig. 1F). Mice that were euthanized also displayed varying levels of abnormal posture (hunching), reduced activity, and fur ruffling, on the day of euthanasia (Supplementary Fig. 2A). For the four mice that were euthanized (Fig. 1E), these mice had very high levels of brain infection (≈8–9 log_10_CCID_50_/mg) (Fig. 1G). At the time of euthanasia, viral titers in the spleen in these mice were below the level of detection (Fig. 1G), consistent with the viremia data. Viral titers in the brain and spleen were assessed exclusively in mice that were humanely euthanized due to disease. Euthanizing healthy mice at corresponding time points was avoided to ensure the generation of a Kaplan–Meier survival curve.

We then infected six-week-old adult C57BL/6J mice with 5 × 10^3^ CCID_50_ (s.c.) of the aforementioned viruses to determine if reduced inoculation dose can increase viremia and neuropenetrance, as has been reported previously^47^. Compared to mice infected with 5 × 10^5^ CCID_50_, viremia in mice infected with 5 × 10^3^ CCID_50_ of virus was significantly higher at later time points for JEV_Nakayama_ (Fig. 1A, day 2–3), JEV_FU_ (Fig. 1B, day 3–4), and MVEV_TC123130_ (Fig. 1D, day 2–3). For JEV_NSW/22_, viremia at day 3 post infection was higher on average but this did not reach statistical significance (Fig. 1C). The viremia area under the curve was significantly higher for JEV_Nakayama_ inoculated with the lower dose (Fig. 1A), but was not significantly different for the other viruses. This coincided with an increase in mortality for JEV_Nakayama_ inoculated at the lower dose, but not for the other viruses (Fig. 1H, I). Viral titers in the brains or spleens of mice that succumbed to infection were not significantly different between inoculum doses (Fig. 1G). JEV_NSW/22_ had a significantly lower viremia compared to JEV_Nakayama_ and JEV_FU_ (Fig. 1J), consistent with significantly lower mortality compared with JEV_Nakayama_ (Fig. 1I), with no C57BL/6J mice infected with JEV_NSW/22_ succumbing to infection (Fig. 1F, I). Overall, this suggests that JEV_NSW/22_ was significantly less virulent in C57BL/6J mice.

### C57BL/6J mice infected with JEV or MVEV led to viral neuroinvasion, apoptosis, and reactive astrogliosis

**Fig. 2:**
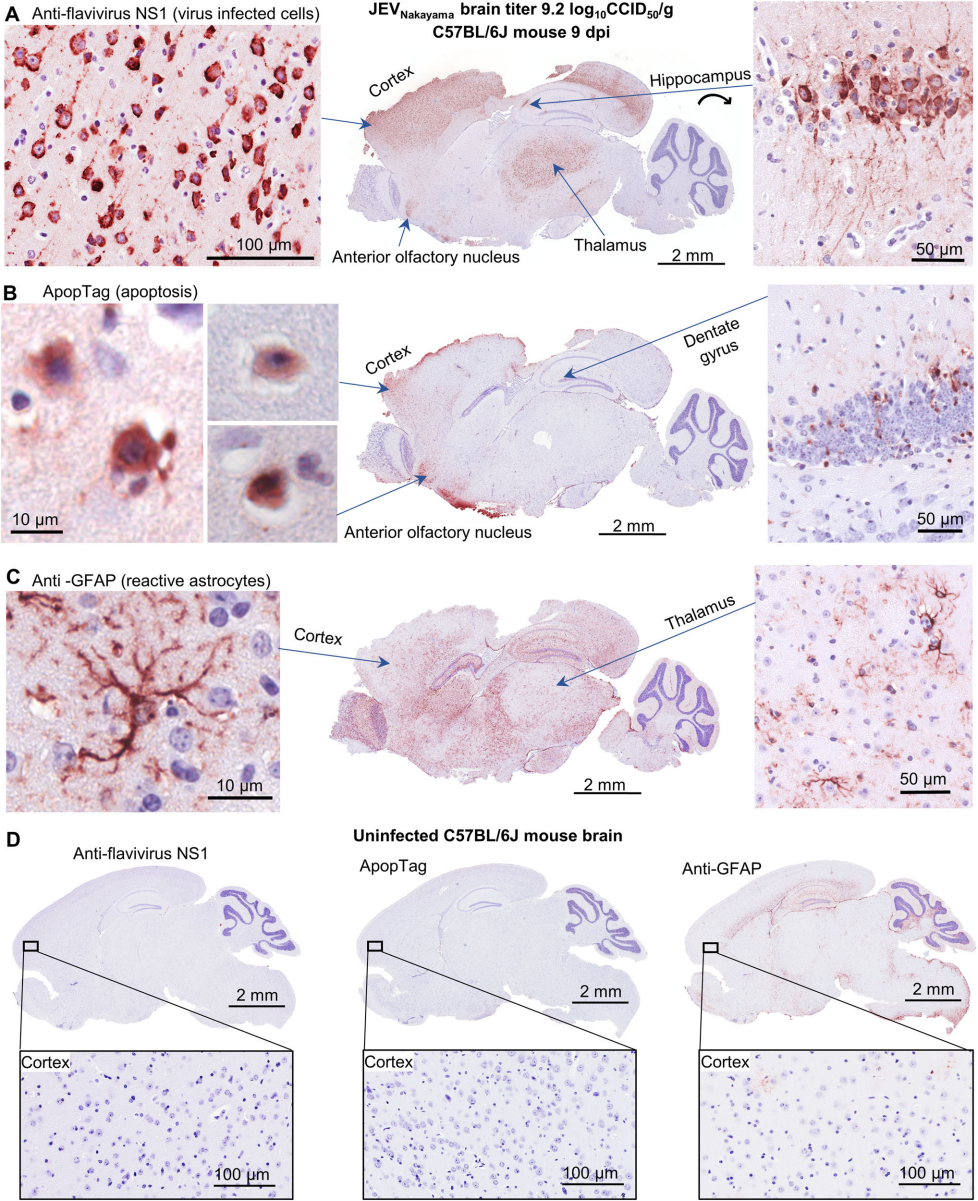
IHC for viral antigen, apoptosis, and reactive astrocytes in JEV-infected C57BL/6J mouse brain. IHC of JEV_Nakayama_ infected brain (required euthanasia on 9 dpi, brain virus titer 9.2 log_10_CCID_50_/g), which is representative of all other C57BL/6J brains from infected mice requiring euthanasia (*n* = 10). **A** Staining for flavivirus NS1 using the 4G4 monoclonal antibody. High magnification images show cells with neuronal morphology in the cortex (left) and hippocampus (right). The latter also shows staining of dendrites and axons (fibrilar patterns above and densely staining cells). 4G4 staining of the brains of the other mice requiring euthanasia (marked by † in Fig. 1E) is shown in Supplementary Fig. 3. **B** ApopTag staining of the same brain as in ‘**A**’. High magnification images show cells with neuronal morphology in the cortex (left) and apoptotic cells in the dentate gyrus (right). ApopTag staining of the brains of the other mice requiring euthanasia is shown in Supplementary Fig. 4. **C** Staining for GFAP, a marker of reactive astrocytes, for the same brain as in ‘**A**’. High magnification images show a typical reactive astrocyte in the cortex (left) and reactive astrocytes in the thalamus (right). GFAP IHC for the brains of the other mice requiring euthanasia is shown in Supplementary Fig. 5. **D** IHC negative controls; uninfected mouse brain stained with 4G4 (left), ApopTag (middle), and anti-GFAP (right).

The brains of C57BL/6J mice were analyzed by immunohistochemistry (IHC) using the pan-flavivirus anti-NS1 antibody (4G4). This antibody detects the viral non-structural protein 1, which is a highly conserved multi-functional protein important for flavivirus replication^48^. Staining was consistently seen in the cortex for JEV and MVEV-infected mice, with staining also seen in the thalamus (Fig. 2A, Supplementary Fig. 3A–C). Prevailing infection of the cerebral cortex and thalamus parallels IHC data from post-mortem human brains^6,49^. In some mice, virus was detected in other brain regions, including the hippocampus, anterior olfactory nucleus (Fig. 2A, JEV_Nakayama_), and caudate putamen (Supplementary Fig. 3B, JEV_FU_). A JEV_FU_-infected mouse that lost ~15% body weight and then recovered (Fig. 1E) had residual virus staining in the cortex at day 29 post infection (Supplementary Fig. 3D), while a mouse that survived infection with minimal weight loss had no detectable viral antigen staining (Supplementary Fig. 3E). As expected, 4G4 staining was associated primarily with cells showing neuronal morphology (Fig. 2A, Supplementary Fig. 3D).

ApopTag staining was evident in areas that were positive for virus staining, particularly in the cortex, and was again associated with cells showing neuronal morphology (Fig. 2B and Supplementary Fig. 4A–C). Apoptotic cells were also evident in the mouse that showed ~15% body weight loss and recovered (Supplementary Fig. 4D). Mice with signs of disease from JEV or MVEV infection (Supplementary Fig. 2) thus showed high levels of viral antigen and apoptosis in the brain. In addition, like in humans, animals can recover from brain infection, despite apoptotic damage, although neurological sequelae may ensue.

Astrocytes are a type of glial cell that provides physical and chemical support to neurons. Astrocytes become activated (reactive astrogliosis) in response to brain pathology, including from JEV infection^50–52^. Reactive astrocytes are characterized by the upregulated expression of glial fibrillary acidic protein (GFAP) and the growth/extension of cellular processes^53^. Brains from mice with signs of disease from JEV or MVEV infection (Supplementary Fig. 2) had significant upregulation of GFAP-positive astrocytes throughout the brain, including the heavily infected areas of the cortex, thalamus and anterior olfactory nucleus (Fig. 2C, Supplementary Fig. 5). GFAP is constitutively expressed in astrocytes of the hippocampus, corpus callosum, and cerebral peduncle^54^, consistent with staining seen in uninfected brains (Fig. 2D, right). Widespread reactive astrogliosis is thus a feature of lethal JEV and MVEV encephalitis in C57BL/6J mice.

### JEV_NSW/22_ is less virulent than JEV_Nakayama_ and JEV_FU_ in Irf7^−/−^ mice

**Fig. 3:**
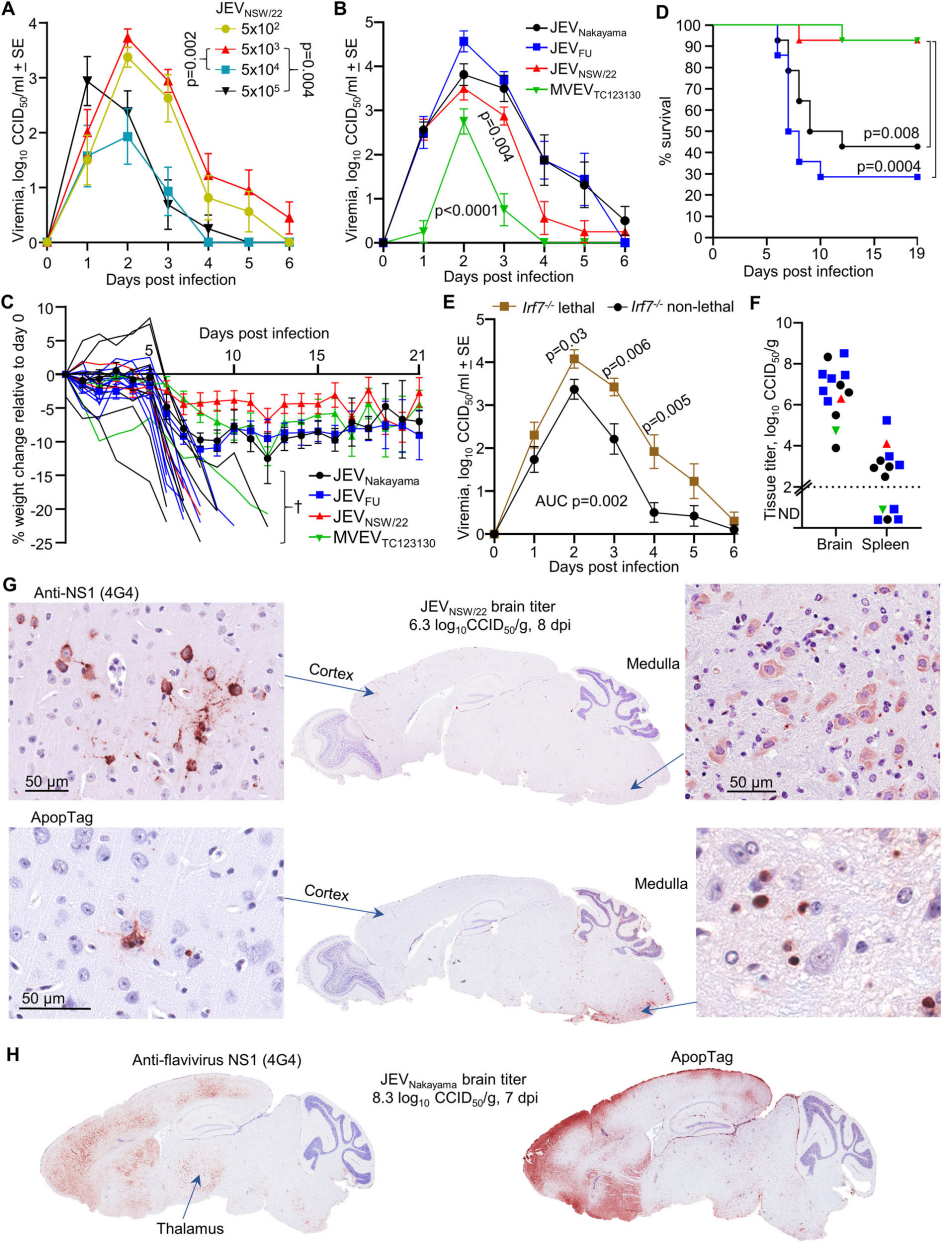
JEV neuroinvasive infection in *Irf7*^*−/−*^ mice. **A** *Irf7*^*−/−*^ mice (15–48 weeks old) were infected s.c. with 5 × 10^5^, 5 × 10^4^, 5 × 10^3^, or 5 × 10^2^ CCID_50_ of JEV_NSW/22_. Data is the mean of *n* = 6 per group and error bars represent standard error. Statistics are a *t* test of area under the curve for the indicated comparisons. **B** *Irf7*^*−/−*^ mice (15–48 weeks old) were infected s.c. with 5 × 10^3^ of the indicated virus (*n* = 8 for each virus). Statistics are a *t* test of area under the curve for JEV_FU_ versus JEV_NSW/22_ or MVEV_TC123130_. **C** Percent body weight change compared to 0 dpi. 20 mice lost >20% body weight or reached a disease score that required euthanized (marked by †) and are plotted individually. The weight change for the remaining mice are shown as means ± SE. Data is from three independent experiments, total *n* = 14 mice per group. **D** Kaplan–Meier plot showing percent survival (*n* = 14 per group, data from three independent experiments). Statistics by log-rank (Mantel–Cox) tests. Symbols as for **C**. **E** Viremias of *Irf7*^*−/−*^ mice infected with either JEV_Nakayama_, JEV_FU_, JEV_NSW/22_, or MVEV_TC123130_ were averaged for mice with non-lethal outcomes (black circles, *n* = 19), versus those with lethal outcomes (brown squares, *n* = 13). Statistics are comparing average viremia of mice with lethal outcomes versus mice with non-lethal outcomes at each timepoint by *t* test or Kolmogorov–Smirnov exact test. *T* test of area under the curve values for this comparison is also shown. **F** Brain and spleen tissue titers for 13 euthanized mice at the time when the criteria for humane euthanasia was met (see Supplementary Fig. 6) (*n* = 5 JEV_Nakayama_, *n* = 6 JEV_FU_, *n* = 1 JEV_NSW/22_, *n* = 1 MVEV_TC123130_). Tissue titers determined by CCID_50_ assay (limit of detection ~2 log_10_CCID_50_/g). **G** IHC of JEV_NSW/22_ infected brain (euthanized on day 8, brain virus titer 6.3 log_10_CCID_50_/g) using 4G4 monoclonal antibody (top) or ApopTag (bottom). High magnification images of cortex (left) and medulla (right). **H** IHC using 4G4 (left) and ApopTag IHC (right) for the JEV_Nakayama_ infected brain with the highest virus titer (euthanized on day 7, brain virus titer 8.3 log_10_CCID_50_/g). IHC images are representative of *n* = 20 brains of mice that succumbed to infection (some other examples are shown in Supplementary Fig. 8).

C57BL/6J mice had a relatively low viremia and low penetrance of lethal brain infection for JEV_NSW/22_ (Fig. 1). A model with a higher penetrance of brain infection is clearly desirable, given encephalitis is the key clinical concern for JEV infections. Interferon regulatory factor 7 knockout (*Irf7*^*−/−*^ mice) were thus chosen as these mice enhanced neuroinvasion for WNV^55^, as well as another arbovirus^30^. We first sought to optimize the inoculation dose for JEV_NSW/22_ in *Irf7*^*−/−*^ mice by comparing 5 × 10^5^, 5 × 10^4^, 5 × 10^3^, and 5 × 10^2^ CCID_50_. The inoculation dose of 5 × 10^3^ CCID_50_ provided the highest viremia area under the curve, which was significantly higher than 5 × 10^4^ and 5 × 10^5^ CCID_50_ inoculation doses (Fig. 3A). The highest infection dose (5 × 10^5^) led to the highest peak viremia on day 1 post infection, but was cleared significantly more quickly than lower infection doses (Fig. 3A, *p* = 0.004). Our data agrees with the concept of an ‘optimal dose’ that is not too high as to excessively stimulate type I IFN, but is high enough to establish a robust viremia^47^. One mouse infected with 5 × 10^3^ CCID_50_ JEV_NSW/22_ succumbed to infection (Fig. 3C, D), while all other mice survived JEV_NSW/22_ infection. An inoculation dose of 5 × 10^3^ CCID_50_ was thus chosen to compare JEV and MVEV strains in *Irf7*^*−/−*^ mice.

JEV_NSW/22_, JEV_Nakayama_, and JEV_FU_ infection of *Irf7*^*−/−*^ mice (dose 5 × 10^3^ CCID_50_) resulted in robust viremias, while MVEV_TC123130_ viremia was significantly lower than JEV (Fig. 3B, *p* < 0.0001). JEV_NSW/22_ had a significantly lower viremia (area under the curve) compared to JEV_FU_ (Fig. 3B, *p* = 0.004). Infection of *Irf7*^*−/−*^ mice with the different JEV isolates and MVEV (*n* = 14 mice per group, *n* = 56 total infected mice) illustrated a mean weight loss of 5–10% for surviving mice across all four groups (Fig. 3C), with the exception of 20 mice that reached ethically defined endpoints for weight loss and/or disease manifestations (Supplementary Fig. 6). Kaplan-Meier curves illustrated significantly higher survival for *Irf7*^*−/−*^ mice infected with JEV_NSW/22_ or MVEV_TC123130_ when compared with JEV_Nakayama_ and JEV_FU_, with only 1/14 JEV_NSW/22_ or MVEV_TC123130_ infected *Irf7*^*−/−*^ mice requiring euthanasia (Fig. 3D). These data are consistent with the significantly lower viremias of JEV_NSW/22_ and MVEV_TC123130_ infected *Irf7*^*−/−*^ mice (Fig. 3B). On average, mice that succumbed to infection (either JEV_Nakayama_, JEV_FU_, JEV_NSW/22_ or MVEV_TC123130_) had a more robust viremia (Fig. 3E), further implicating a higher viremia in a higher chance of lethal neuropenetrance in *Irf7*^*−/−*^ mice.

Brain titers in euthanized *Irf7*^*−/−*^ mice were ~4–9 log_10_CCID_50_/gram, while spleen titers were <2–5 log_10_CCID_50_/g (Fig. 3F). IHC staining for viral antigen confirmed JEV infection in the brains of *Irf7*^*−/−*^ mice, with reduced levels of staining reflecting lower brain titers (Fig. 3G, H, Supplementary Fig. 7). Viral antigen staining was consistently present in the cortex, with staining also seen in the medulla, thalamus, and hippocampus (Fig. 3G, H, Supplementary Fig. 7). Brain regions showing ApopTag staining broadly overlapped with staining for viral antigen (Fig. 3G, H). Reactive astrogliosis was also evident (Supplementary Fig. 5). Overall, this suggests that JEV infection of *Irf7*^*−/−*^ mice produced a consistent viremia, with mice showing brain infection and apoptotic damage.

### JEV_NSW/22_ and JEV_FU_ are more sensitive to type I IFN compared to JEV_Nakayama_ and MVEV_TC123130_

**Fig. 4:**
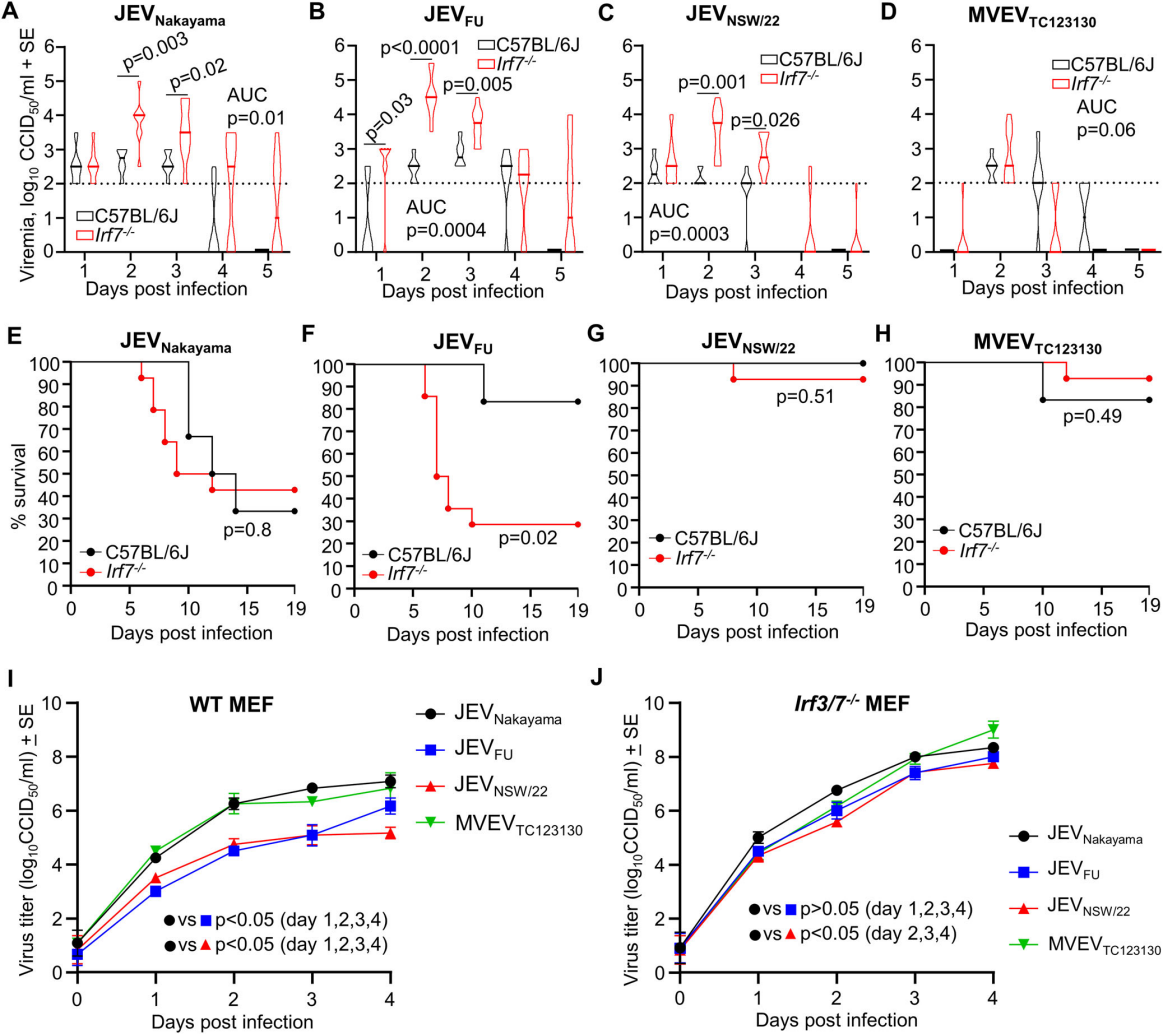
Comparisons of viremia and survival between C57BL/6J versus *Irf7*^*−/−*^ mice, and comparison of virus replication in wild-type versus *Irf3/7*^*−/−*^ mouse embryonic fibroblasts (MEFs). C57BL/6J or *Irf7*^*−/−*^ mice were infected with 5 × 10^3^ CCID_50_ of JEV_Nakayama_, JEV_FU_, JEV_NSW/22_, or MVEV_TC123130_. Data shown in A-H are a reanalysis of data presented in Fig. 1A–D, Fig. 1I, Fig. 3B, D. **A**–**D** Female ≈6 week old C57BL/6J mice (black) or *Irf7*^*−/−*^ mice (red) were infected s.c. with 5 × 10^3^ CCID_50_ of the indicated viruses. Violin plots for *n* = 6 (C57BL/6 J) or *n* = 8 (*Irf7*^*−/−*^) per group over 5 days is shown. The horizontal line within the violin plot represents the median. Limit of detection is 2 log_10_CCID_50_/ml of serum. Statistics represent *t* test or Kolmogorov–Smirnov exact test at the indicated timepoint or for area under the curve (AUC) values (see statistics methods section). **E**–**H** Kaplan-Meier plot showing percent survival for C57BL/6J mice (black line, *n* = 6) and Irf7^−/−^ mice (ref line, *n* = 14) infected with 5 × 10^3^ CCID_50_ of the indicated virus. Statistics by log-rank (Mantel–Cox) tests. **I** Wild-type or **J** *Irf3/7*^*−/−*^ MEFs were infected with JEV_Nakayama_ (black circles), JEV_FU_ (blue squares), JEV_NSW/22_ (red triangles), or MVEV_TC123130_ (green downward triangles) at MOI 0.1. Virus titer in the culture supernatant was monitored over 4 days. Data is the mean of two independent experiments with a total of *n* = 6 replicates per group. Error bars represent standard error. Statistics are by *t* test or Kolmogorov–Smirnov exact test for the indicated comparisons.

Viremia and survival were compared between C57BL/6J and *Irf7*^*−/−*^ mouse strains infected with 5 × 10^3^ CCID_50_ virus to determine whether differences in viremia may be associated with the differences in viral neuropenetrance and survival. Area under the curve analyses showed a significantly increased/prolonged viremia in *Irf7*^*−/−*^ mice compared to C57BL/6J mice for all JEV strains (Fig. 4A–C). However, MVEV_TC123130_ viremia was not significantly improved in *Irf7*^*−/−*^ mice (Fig. 4D). Kaplan–Meier survival curves indicated that JEV_FU_ had a significantly increased mortality in *Irf7*^*−/−*^ mice compared to C57BL/6J mice (Fig. 4F), while there was no significant difference between mouse strains for the other viruses (Fig. 4E, G–H).

Differences in sensitivity to type I IFN between virus strains may explain the different outcomes in C57BL/6J versus *Irf7*^*−/−*^ mice. To examine type I IFN sensitivity in the absence of *in vivo* adaptive immune responses, mouse embryonic fibroblasts (MEFs) with (wild-type) and without (*Irf3/7*^*−/−*^) functional type I IFN production were infected with JEV_Nakayama_, JEV_FU_, JEV_NSW/22_ and MVEV_TC123130_. In wild-type MEFs, JEV_FU_ and JEV_NSW/22_ had significantly lower virus replication kinetics compared to JEV_Nakayama_ and MVEV_TC123130_ (Fig. 4I). In *Irf3/7*^*−/−*^ MEFs, the difference in replication between virus strains was diminished, with JEV_FU_ not significantly different to JEV_Nakayama_ (Fig. 4J). This suggests that JEV_FU_ and JEV_NSW/22_ were more sensitive to type I IFN compared to JEV_Nakayama_ and MVEV_TC123130_, consistent with an increase in mortality in *Irf7*^*−/−*^ mice (partially defective type I IFN responses) compared to C57BL/6J mice for JEV_FU_ but not for JEV_Nakayama_ or MVEV_TC123130_. Although not statistically significant, this trend was also observed for JEV_NSW/22,_ which caused mortality in one *Irf7*^*−/−*^ mouse, compared to zero C57BL/6J mice (Fig. 4G).

### Ifnar^−/−^ mice are a model of lethal JEV viremia without neuroinvasion

**Fig. 5:**
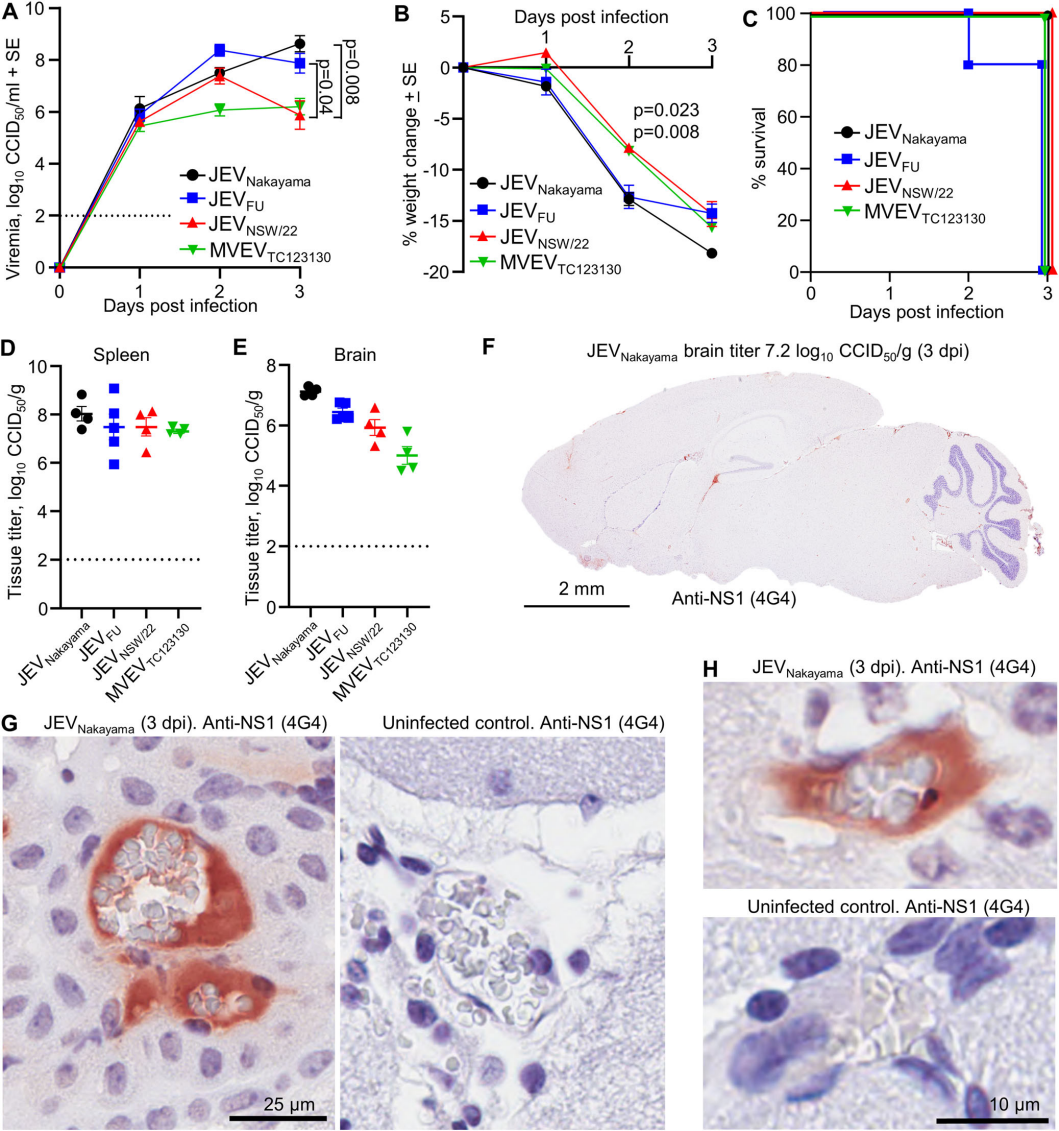
JEV and MVEV lethal viremia in *Ifnar*^*−/−*^ mice. Female *Ifnar*^*−/−*^ mice (9–24 week old) were infected s.c. with 5 × 10^5^ CCID_50_ of the indicated virus (*n* = 4 for JEV_Nakayama_, JEV_NSW/22_ and MVEV_TC123130_, and *n* = 5 for JEV_FU_). **A** Mean viremia determined by CCID_50_ assay (limit of detection 2 log_10_CCID_50_/ml). Statistics by *t* tests. **B** Mean percent body weight change compared to 0 dpi. Statistics 2 dpi JEV_NSW/22_ versus JEV_Nakayama_ (Kolmogorov–Smirnov test, *p* = 0.023) and for JEV_NSW/22_ versus JEV_FU_ (*t* test, *p* = 0.008). **C** Kaplan–Meier plot showing percent survival. **D** Viral tissue titers in spleens harvested at euthanasia (3 dpi for all mice except for 1 JEV_FU_ mouse at 2 dpi), determined by CCID_50_ assay (limit of detection ~2 log_10_ CCID_50_/g). **E** Viral titers in brains harvested at euthanasia. **F** IHC staining for flavivirus NS1 using the 4G4 monoclonal antibody. The brain shown was infected with JEV_Nakayama_ (titer 7.2 log10 CCID50/g, 3 dpi). Staining was representative of all other JEV brains. **G**, **H** High magnification images from F showing NS1 staining in blood vessels surrounding the pale gray, biconcave shaped, red blood cells.

As JEV_NSW/22_ did not lead to fatal neuroinvasion in C57BL/6J mice, and fatal neuroinvasion in Irf7^−/−^ mice was rare, type I interferon receptor knockout (*Ifnar*^*−/−*^) mice were evaluated. *Ifnar*^*−/−*^ mice have been used extensively in studies of pathogenic flaviviruses such as Zika virus (ZIKV), West Nile virus (WNV), and Yellow fever virus (YFV), and generally provide a robust viremia and a lethal outcome^23–25,35^. Adult *Ifnar*^*−/−*^ mice were infected with 5 × 10^5^ CCID_50_ of JEV_Nakayama_, JEV_FU_, JEV_NSW/22_, or MVEV_TC123130_ via s.c. injection. A robust viremia developed, reaching 6–8 log_10_CCID_50_/ml of serum by 2 dpi (Fig. 5A). JEV_NSW/22_ infected mice had ≈2 log lower viremia compared to JEV_Nakayama_ and JEV_FU_ on 3 dpi (Fig. 5A). Mice displayed a rapid loss of body weight, which was slightly delayed for JEV_NSW/22_ and MVEV_TC123130_ at 2 dpi (Fig. 5B), requiring euthanasia 2/3 dpi (Fig. 5C). Mice infected with any of the JEV strains also displayed varying levels of abnormal posture (hunching), reduced activity, and fur ruffling (Supplementary Fig. 8A).

Consistent with the robust viremia (Fig. 5A), spleen tissue titers reached ~6–9 log_10_CCID_50_/g at 2–3 dpi (Fig. 5D). Similar levels were seen in brains (Fig. 5E); however, 4G4 staining illustrated that brain cells were not infected (Fig. 5F). Viremia and brain titers correlated strongly (Supplementary Fig. 8B), arguing that the brain titers (Fig. 5E) likely arose from virus in the blood vessels of the brain. Interestingly, anti-flavivirus NS1 antibody staining was clearly present in blood vessels (Fig. 5G, H), with extracellular JEV NS1 also found in serum of JEV patients^56^. Secreted NS1 from JEV-infected cells is reported to increase vascular leakage and may contribute to mortality^57^, although overt vascular leakage of NS1 was not evident in IHC, with lack of type I IFN signaling potentially involved^58,59^. Neither apoptosis (Supplementary Fig. 8C), nor significantly increased staining for reactive astrocytes (Supplementary Fig. 5, *Ifnar*^*−/−*^) was apparent for the brains of JEV-infected *Ifnar*^*−/−*^ mice. Therefore, *Ifnar*^*−/−*^ mice infected with JEV or MVEV represent a model of lethal viremia without viral neuroinvasion.

### JEV causes severe histological lesions in C57BL/6J and Irf7^−/−^ mouse brains

**Fig. 6:**
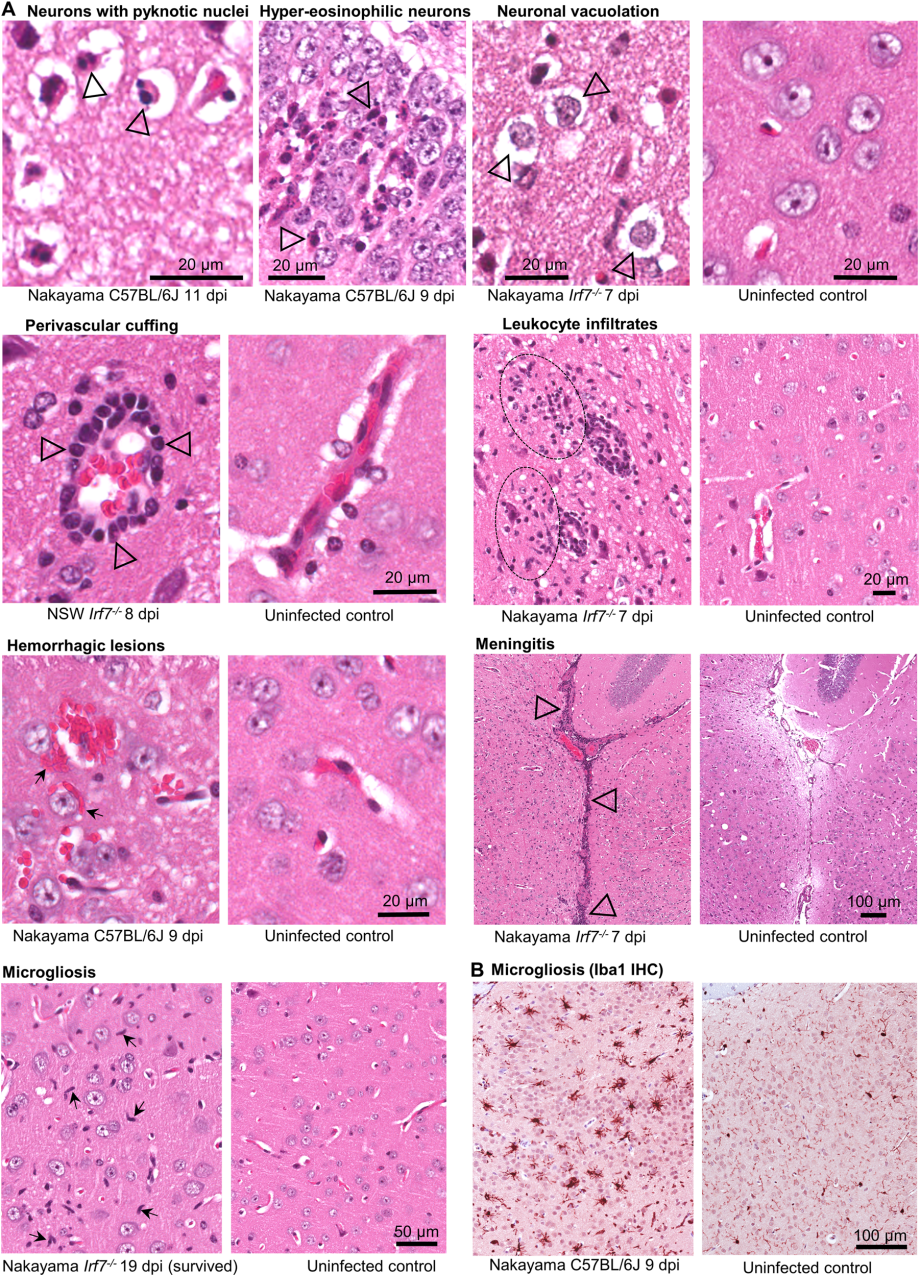
Histological lesions in brains of JEV-infected mice. **A** Examples of indicated lesions stained with H&E. Degeneration of neurons indicated by (i) pyknotic nuclei (black unfilled arrowheads indicating condensation and fragmentation of nuclei, staining dark blue), and (ii) hyper-eosinophilic cytoplasm of degenerating neurons in the cornus ammonis of the hippocampus (area also staining for viral antigen Supplementary Fig. 10). Neuronal vacuolation indicated by fluid accumulation around the neurons (arrowheads). Perivascular cuffing is indicated by leukocytes aggregating in blood vessels (arrowheads). Leukocyte infiltrates (extravascular) are indicated by dashed ovals. Hemorrhagic lesions are indicated by extravascular red blood cells (arrows). Microgliosis is indicated by accumulation of microglia, which have elongated rod-shaped nuclei (arrows). Meningitis is indicated by accumulation of leukocytes around the meninges (arrowheads). Images of uninfected controls accompany each image(s) of lesions. Histology scores for all mouse brains are shown in Supplementary Figs. 9, 10. **B** IHC using anti-Iba1, a microglial marker.

H&E staining of JEV-infected mouse brains revealed a series of histopathological lesions, predominantly neuronal degeneration with pyknotic nuclei (often associated with apoptosis), neuronal vacuolation, perivascular cuffing, leukocyte infiltrates, hemorrhage, meningitis and microgliosis (Fig. 6A). IHC for the microglial marker, Iba1, confirmed the presence of microgliosis (Fig. 6B).

The presence of H&E lesions was scored for the different JEV isolates and mouse strains (Supplementary Fig. 9). C57BL/6J and *Irf7*^*−/−*^ mice that were euthanized at the time when viral neuroinvasion led to the criteria for humane euthanasia being met (full score cards provided in Supplementary Figs. 2 and 7) had a broadly similar incidence of the aforementioned lesions, whereas only hemorrhage was seen in *Ifnar*^*−/−*^ mice (Supplementary Fig. 9, Acute). Leukocyte infiltration was quantified by calculating the ratio of nuclear/cytoplasmic staining, as leukocytes have a higher ratio of nuclear to cytoplasmic staining^33^. This confirmed that C57BL/6J and *Irf7*^*−/−*^ mice that succumbed to infection had significantly increased leukocyte infiltrates (Supplementary Fig. 9B). For C57BL/6J and *Irf7*^*−/−*^ mice that survived and were euthanized later (nominally chronic phase), only hemorrhage was consistently observed (Supplementary Fig. 9, Chronic).

Lesions in C57BL/6J and *Irf7*^*−/−*^ mouse brains were associated with areas of high virus infection, most prominently in the cortex (Supplementary Fig. 10); however, lesions were also found in other areas of the brain including where there was minimal viral antigen staining (Supplementary Fig. 11A). Some mice that lost >8% body weight and then recovered had persistent lesions detectable at the latest time point sampled (up to day 32 for C57BL/6J and day 21 for *Irf7*^*−/−*^) (Supplementary Fig. 11B), consistent with persistent neurological sequelae in humans that survive infection. Mice that did not lose any body weight did not show any overt brain lesions at the later time points, indicating that brain infection causing weight loss is associated with persistent lesions.

Overall, the lesions in C57BL/6J and *Irf7*^*−/−*^ mouse brains are consistent with H&E detectable lesions in post-mortem human JEV-infected brains^6,50,60–62^. To provide additional potential insights into the nature of the cellular infiltrates (e.g., perivascular cuffing and leukocyte infiltrates, Fig. 6), immune cell type abundance estimates were obtained from reanalyzed^63^ publically available JEV-infected mouse brain RNA-Seq expression data^64^ using SpatialDecon^65^. The dominant cell types identified were CD4 T cells, NKT cells, monocytes/macrophages, neutrophils, innate lymphoid cells, T regs, and CD8 T cells, with an increase in microglia cells also identified (Supplementary Fig. 12). GFAP was significantly upregulated 5.4-fold by infection (Supplementary Table 3, log_2_FC 2.4), consistent with the GFAP IHC (Fig. 2C) and reactive astrogliosis seen herein. Iba1 (Aif1) was also significantly upregulated 5.5-fold (Supplementary Table 3, log_2_FC 2.5), consistent with the H&E and IHC (Fig. 6) and microgliosis observed in our study.

### Productive replication of JEV in human cortical brain organoids

**Fig. 7:**
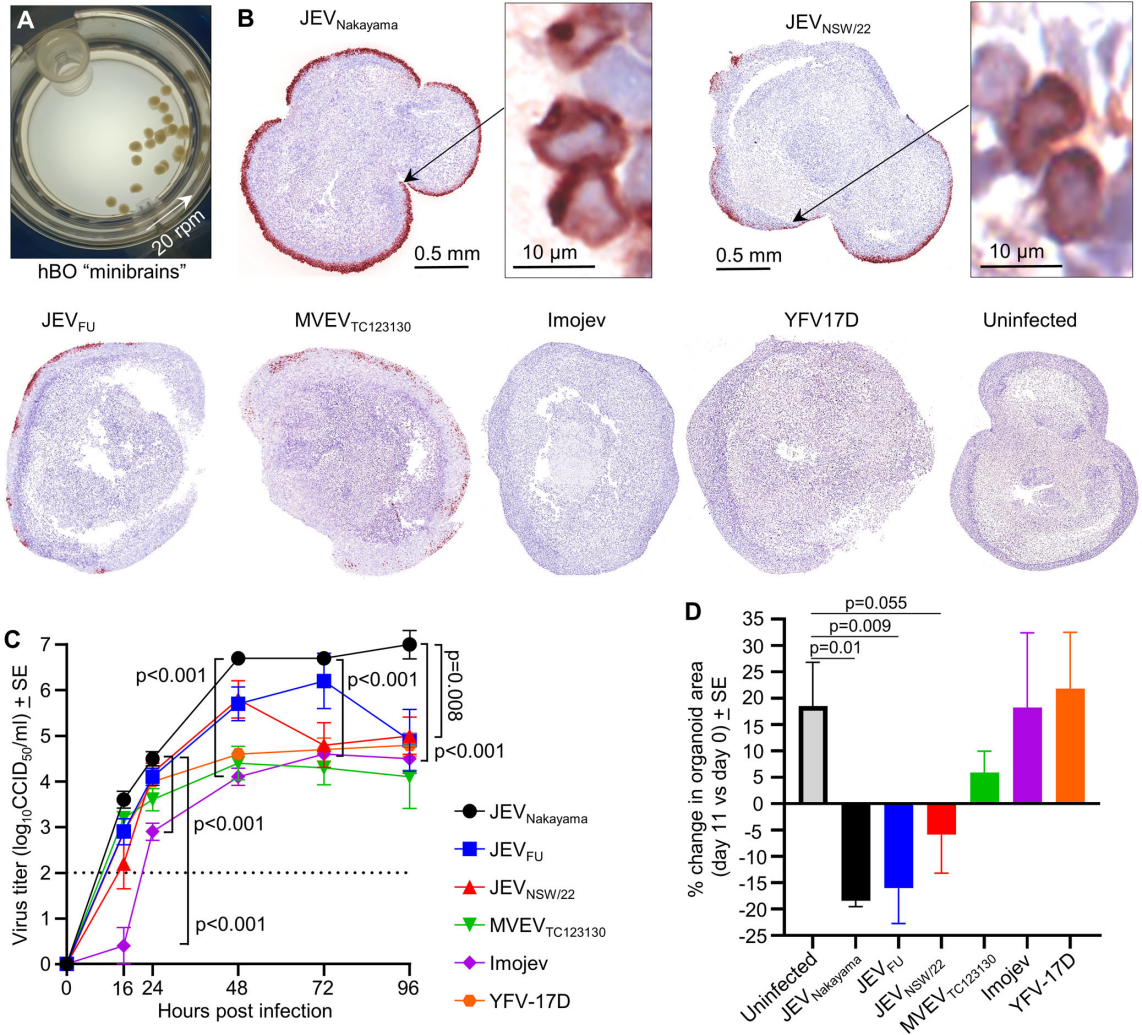
Infection of human cortical brain organoids (hBOs). **A** Photograph of “mini-brains” cultured in a rotating CelVivo Clinostar incubator. **B** IHC of viral antigen (4G4) for hBOs at 4 dpi. Images are representative of *n* = 4 hBOs for each group. Magnified images of sparse IMOJEV and YFV 17D infected cells are shown in Supplementary Fig. 12A. **C** Viral growth kinetics up to 4 dpi determined by CCID_50_ assays of culture supernatants at the indicated hours post infection; limit of detection is 2 log_10_CCID_50_/ml. At all time points JEV_Nakayama_ vs. IMOJEV, and at 96 h JEV_Nakayama_ vs. JEV_NSW/22_ were significant (*t* tests, *n* = 5 organoids per group). **D** Mean percentage change in organoid area at 11 dpi vs. 9 dpi for each organoid (*n* = 8 for uninfected and MVEV_TC123130_, otherwise *n* = 4). Statistics are by Kolmogorov–Smirnov exact test for uninfected versus JEV_Nakayama_, and *t* test for uninfected versus JEV_FU_ or JEV_NSW/22_.

Human cortical brain organoids (hBOs) have been used to investigate the pathogenesis of flaviviruses, including JEV and ZIKV^23,66,67^. To determine JEV replication capacity in neuronal cells and the ensuing cytopathogenicity, hBOs were used as an alternative to intracranial injection of mice. We thus generated ~30-day-old hBOs from adult human dermal fibroblast (HDFa) cells, growing the hBOs in a CelVivo Clinostar incubator as described^37^ (Fig. 7A). hBOs were infected with 10^5^ CCID_50_ of the indicated JEV and MVEV isolates, as well as (i) the IMOJEV chimeric virus vaccine (previously called ChimeriVax-JE) comprising the prME genes of the attenuated JEV_SA14-14-2_ strain on the YFV 17D backbone^38,68^ and (ii) the Yellow Fever live attenuated vaccine strain (YFV 17D)^25^, with wild-type YFV infection^69^, and occasionally YFV 17D vaccination^70^, able to cause neuropathology. hBOs were fixed in formalin 4 dpi, and IHC was undertaken using the anti-NS1 monoclonal antibody 4G4^25^. JEV_Nakayama_-infected hBOs showed the most pronounced viral antigen staining, with JEV_NSW/22_, JEV_FU_, and MVEV_TC123130_ also showing clear staining (Fig. 7B). Viral antigen was primarily localized to the outer surface of the organoids (Fig. 7B), where cells are in direct contact with the culture medium. Viral antigen staining for IMOJEV and YFV 17D infected hBOs was also seen but was sparse (Fig. 7B and Supplementary Fig. 13A).

All viruses were able to productively infect hBOs, with JEV_Nakayama_ infection generating higher viral titers than IMOJEV at all time points (Fig. 7C, *p* < 0.001). By 96 h JEV_Nakayama_ titers were also significantly higher than those seen for JEV_NSW/22_ (Fig. 7C).

Over 11 days, uninfected hBOs grew in circumference by ~20% (Fig. 7D, Uninfected). hBOs infected with JEV isolates shrank in circumference by ~5 to 15% by 11 dpi, although when compared with uninfected organoids, this only approached significance for JEV_NSW/22_ (Fig. 7D; Supplementary Fig. 13B, C). hBOs infected with MVEV_TC123130_, IMOJEV and YFV 17D infected hBO did not shrink significantly when compared with uninfected controls, although the MVEV_TC123130_ data suggested a marginal reduction in circumference (Fig. 7C; Supplementary Fig. 13C). These data (Fig. 7D) reflect differences in viral replication (Fig. 7C) and/or viral protein immunohistochemistry (Fig. 7B) and likely reflect virus-induced CPE.

All viruses replicated productively in a human neural progenitor cell line, RENcell VM, with all viruses except IMOJEV causing fulminant CPE (Supplementary Fig. 14A, B). Although Vero E6 and BHK-21 cells are widely used for flavivirus research, CPE induced by JEV_NSW/22_ was considerably less pronounced in these cells (Supplementary Fig. 14C, D).

### Human post-IMOJEV vaccination sera neutralizes JEV and MVEV with titers related to envelope protein amino acid conservation

**Fig. 8:**
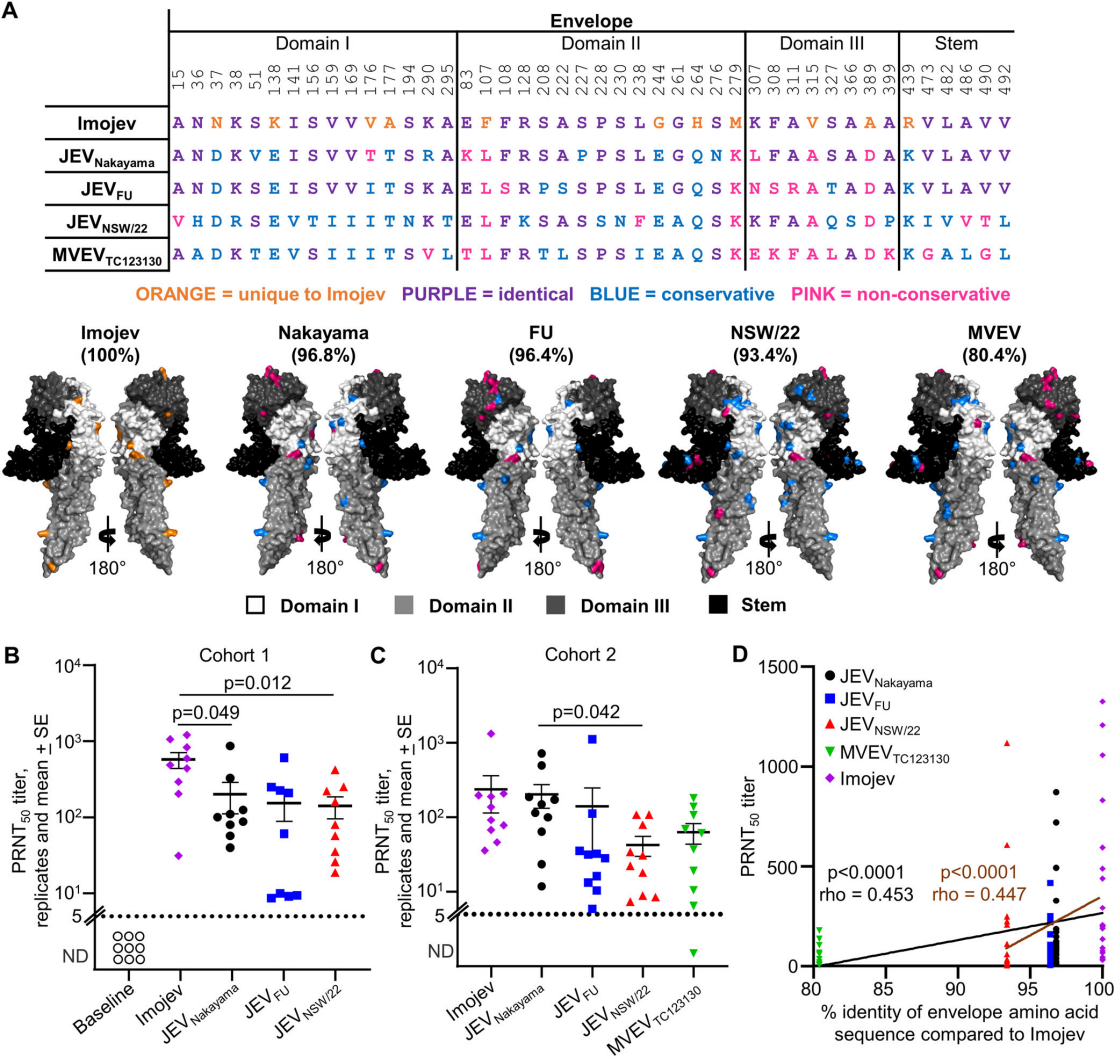
Human post-IMOJEV vaccination sera neutralizes JEV and MVEV with titers related to envelope protein amino acid conservation. **A** Envelope protein (domains I, II, III, and STEM) amino acid sequences for IMOJEV, JEV_Nakayama_, JEV_FU_, JEV_NSW/22_, and MVEV_TC123130_ (refer to Supplementary Fig. 1 for GenBank accession numbers). Sequences for isolates were aligned using MEGA-X and the ClustalW plugin with default parameters. Coloring indicates amino acid category compared to IMOJEV (orange = unique to IMOJEV, purple = identical, blue = conservative amino acid difference, pink = non-conservative amino acid difference^43^). Crystal structure of JEV envelope (PDB: 5WSN) with amino acid differences for JEV_Nakayama_, JEV_FU_, JEV_NSW/22_, and MVEV_TC123130_ compared to IMOJEV colored as described in the table. Percentages indicate percent sequence identity relative to IMOJEV. **B** Human serum taken at day 0 and day 28 post-IMOJEV vaccination (*n* = 9, cohort 1) was used in plaque reduction neutralization assays against JEV_Nakayama_, JEV_FU_, and JEV_NSW/22_, and the plaque reduction neutralization 50% titer (PRNT_50_) was calculated. Mean and standard errors are shown. Statistics are paired *t* test comparing IMOJEV with JEV_Nakayama_ or JEV_NSW/22_. **B** Human serum taken 2–12 months post-IMOJEV vaccination (*n* = 10, cohort 2) was used in plaque reduction neutralization assays against JEV_Nakayama_, JEV_FU_, JEV_NSW/22_, and MVEV_TC123130_ and the plaque reduction neutralization 50% titer (PRNT_50_) was calculated. Mean and standard errors are shown. Statistics are paired *t* test comparing JEV_Nakayama_ with JEV_NSW/22_. **D** PRNT_50_ titers in ‘**B**’ and ‘**C**’ plotted against percentage envelope protein amino acid identity in ‘**A**’. Curve fit is shown, and statistics calculated by Spearman correlation with p and rho values shown (black line represents all data, brown line excludes MVEV_TC123130_ from analysis).

IMOJEV is one of two JEV vaccines available in Australia. The IMOJEV prME genes are derived from the genotype 3 JEV_SA14-14-2_^38,68^ strain, which was attenuated via extensive in vitro and in vivo passaging (Supplementary Fig. 1). Most flavivirus-neutralizing antibodies recognize epitopes on the envelope protein, particularly in the putative receptor binding domain III^71^. JEV_Nakayama_ and the JEV_SA14-14-2_ component of the IMOJEV vaccine both belong to genotype 3, but JEV_Nakayama_ has 96.8% envelope protein amino acid identity to IMOJEV (Fig. 8A). JEV_FU_ has 96.4% envelope protein identity, while JEV_NSW/22_ has drifted further from the genotype 2 and 3 strains with 93.4% envelope protein identity (Fig. 8A). MVEV_TC123130_, which is the closest phylogenetically related flavivirus to JEV (Supplementary Fig. 1B), has 80.4% envelope amino acid identity (Fig. 1A). Alignment of the envelope amino acid differences for these strains compared to IMOJEV reveal that a disproportionate number of the non-conservative changes were in domain III (Fig. 8A).

Serum-neutralizing antibodies post-vaccination are currently viewed as the best measurable correlate of vaccine protection for JEV^72^. To determine if the Australian outbreak genotype 4 JEV_NSW/22_ is neutralized by antibodies produced in response to IMOJEV vaccination, serum was taken from two human cohorts. Cohort 1 was serum collected from individuals at approximately 28 days post-vaccination, while cohort 2 serum was collected at variable times post-vaccination (2 months to 1 year). Ethical approvals for cohort 1 serum allowed for neutralization assays against the IMOJEV vaccine itself, JEV_Nakayama_, JEV_FU_, and JEV_NSW/22_ (Fig. 8B), while ethical approval for cohort 2 additionally allowed for neutralization assays against MVEV_TC123130_ (Fig. 8C). All serum samples from both cohorts had a measurable 50% neutralization titer (PRNT_50_) titer against JEV_Nakayama_, JEV_FU_, and JEV_NSW/22_ (Fig. 8B, C), indicating that seroconversion (defined as detectable neutralizing antibodies >10^1^ PRNT_50_ titer) provided neutralizing antibodies that cross-react between JEV genotypes 2, 3, and 4. In cohort 1, PRNT_50_ titers were significantly lower against JEV_Nakayama_ and JEV_NSW/22_ compared to against IMOJEV (Fig. 8A), suggesting key antigenic differences from the vaccine. In cohort 2, PRNT_50_ titers against JEV_Nakayama_ were not significantly different compared to IMOJEV, however PRNT_50_ titers were significantly lower for JEV_NSW/22_ (Fig. 8C). Similar conclusions were drawn when the raw percentage of plaque neutralization at a high serum dilution (1:160) was compared between virus strains (Supplementary Fig. 15). With PRNT_50_ data from both cohorts combined, the percentage amino acid identity of the envelope protein compared to the IMOJEV vaccine significantly correlated with PRNT_50_ titers (Fig. 8D, black line). The significance (p-value) and the correlation coefficient (rho), were similar when the same analysis was conducted, excluding the PRNT_50_ data for MVEV_TC123130_ (Fig. 8D, brown line). Overall, our data indicates that IMOJEV vaccination provided neutralizing antibodies against JEV_NSW/22_ in all individuals, but the level of cross-neutralization was related to the conservation in envelope protein amino acid sequences.

## Discussion

Herein we provide a comprehensive *in vivo* and *in vitro* characterization of the genotype 4 JEV_NSW/22_ isolate from the recent Australian outbreak and illustrate mouse models of infection and rare CNS neuropathological manifestations that recapitulate many aspects of human and primate disease^6,49–52,73^. The capacity of JEV_NSW/22_ to cause lethal neuroinvasive infection in mice was significantly diminished compared to JEV_Nakayama_ and JEV_FU_, with only one *Irf7*^*−/−*^ mouse succumbing to JEV_NSW/22_ infection out of 63 infected C57BL/6J or *Irf7*^*−/−*^ mice. Such rare lethal neuroinvasion recapitulates what is seen in humans, with ~1 in 750 infections causing fatality^2,3^. Serosurvey data, albeit limited, suggests the ratio of human symptomatic to asymptomatic infections is not particularly different for JEV_NSW/22_. To date, 45 clinical cases have been notified for the recent outbreak in Australia^74^, with serosurveys in Victoria (*n* = 820 participants) and New South Wales (NSW) (*n* = 917 participants) reporting 3.3% and 8.7% of participants as seropositive for JEV, respectively^75,76^. Although somewhat dated, population data for the primary recruitment locations for the serosurveys is available from Australian Bureau of Statistics 2016, with Victorian recruitment locations providing a population total of 160,294 (Mildura, Lockington, Shepparton, Cobram, Yarrawonga, Rutherglen, Wodonga, Wangaratta, Rochester), and NSW locations a total of 68,431 (Balranald, Corowa, Dubbo, Griffith, Temora). As [0.033 × 160,294] + [0.087 × 68,431]/250 = 45, the serosurvey data is consistent with the expected symptomatic to asymptomatic ratio of ≈1 in 250 for JEV and thus provides no compelling evidence for overt virulence differences for JEV_NSW/22_ in human populations.

Our *Irf7*^*−/−*^ mouse model of JEV_NSW/22_ provides for a more robust viremia, and a slightly higher chance of lethal neuroinvasive infection. Increased lethal neuropenetrance in *Irf7*^*−/−*^ mice was associated with prolonged viremia, possibly via increased inflammation-driven blood-brain barrier breakdown as a result^5,77^. The use of *Irf7*^*−/−*^ mice to increase lethal neuroinvasive infection compared to C57BL/6J was only suitable for JEV_FU_ and JEV_NSW/22_. This was likely due to higher sensitivity to type I IFN for these isolates, demonstrated using MEF cells, compared to JEV_Nakayama_ and MVEV_TC123130_. The partially defective type I IFN responses in *Irf7*^*−/−*^ mice^30^ thus provide a benefit for JEV_FU_ and JEV_NSW/22_ lethal neuropenetrance, but not for JEV_Nakayama_ or MVEV_TC123130_. When type I IFN responses were completely defective (*Ifnar*^*−/−*^ mice and *Irf3/7*^*−/−*^ MEFs), differences between virus replication and/or lethality were minimal. Overall, these results suggest that JEV_NSW/22_ may be more sensitive to, or less able to suppress, type I IFN responses. Inhibition of type I IFN responses is mediated by NS5 for inhibition of STAT2 and NS3^78^ and subgenomic flavivirus RNA (sfRNA)/NS5^23^ for inhibition of STAT1. Although the latter likely operates for WNV in mice^79^ and is involved in promoting apoptosis^23^, the efficiency of these systems during JEV infection of humans and mice remains to be determined. sfRNA is derived from the 3’UTR^23^, where JEV_NSW/22_ does show a small number of nucleic acid changes, but which are unlikely to affect sfRNA production (Supplementary Table 2). Mouse models of flavivirus pathogenesis frequently use *Ifnar*^*−/−*^ mice^25,80,81^ as flaviviruses often replicate poorly in wild-type mice as the ability of NS5 to suppress the antiviral type I IFN responses in humans often fails to operate in mice^82^. We show herein that *Ifnar*^*−/−*^ mice are not a good model for JEV neuropathology, as they reach ethically defined endpoints before brain infection can occur. However, *Ifnar*^*−/−*^ mice are a good model of robust and lethal viremia, which may provide a useful and stringent model for vaccine testing.

JEV_NSW/22_ does not contain known attenuating mutations that would explain its reduced virulence in mice. There are 9 amino acids in the IMOJEV vaccine envelope gene that have been associated with attenuated virulence; F107, K138, V176, A177, G244, H264, M279, V315, and R439^83–93^. At these positions, JEV_Nakayama_, JEV_FU_, JEV_NSW/22_, and MVEV_TC123130_ all have the same amino acids (L107, E138, T177, E244, Q264, K279, A315, and K439), except for position 176 where JEV_Nakayama_ has T176, and JEV_FU_, JEV_NSW/22_, and MVEV_TC123130_ have I176 (Supplementary Table 2). JEV_NSW/22_ retains E at position 138, and this amino acid has been identified by several studies as a principal virulence determinant^94–98^, with a role in neuronal cell binding hypothesized^42^. YFV 17D has a valine (V) at this residue, possibly contributing to reduced virulence^99^. NS1 and NS2A have been implicated in JEV virulence^92^. Among the six changes in NS1 associated with attenuation of virulence are R147H and R339M^92^, of which H147 is present in both JEV_Nakayama_ and JEV_NSW/22_, with K339 (a conserved substitution for R) present in JEV_NSW/22_. JEV non-structural protein 4B (NS4B) alone can induce apoptosis and encephalitis^100^, however, NS4B is completely conserved between JEV_Nakayama_, JEV_FU_, and JEV_NSW/22_ (Supplementary Table 2). prM has been reported to influence the virulence of ZIKV^80^ and JEV virulence in mice^101^. JEV_NSW/22_ has a number of unique changes in prM (Supplementary Table 2), although their functional implications remain unclear. JEV_NSW/22_ also has an additional N-linked glycosylation site at position 175 in NS1 that is lacking in JEV_Nakayama_ and JEV_FU_ (Supplementary Table 2). However, this N-linked glycosylation site is reported to increase WNV virulence in wild-type mice^102,103^, a trend not seen in our JEV data (Fig. 1F). Thus, JEV_NSW/22_ shows no obvious sequence characteristics that can be readily associated with the reduced virulence in mice, and mutagenesis experiments would be required to fully understand these differences.

All human participants were vaccinated with the IMOJEV vaccine-induced neutralizing antibodies to JEV_NSW/22_, suggesting that this vaccine, which is available in Australia, is likely to afford some protection against Australian outbreak genotype 4 JEV. However, the divergence of envelope protein amino acid sequences from that of the IMOJEV vaccine affected the PRNT_50_ titers, although it is unclear how this may translate to the impact on vaccine efficacy, especially given that in vitro neutralization assays do not capture the full range of protective mechanisms mobilized in vivo^104^. Nonetheless, this provides a strong rationale for the development of updated JEV vaccines that use antigen sequences from currently circulating JEV strains, such as genotype 4 in Australia^14^, genotype 5 in Republic of Korea^12^, and genotype 1 in most other areas of South East Asia^105^. IMOJEV vaccination also produced neutralizing antibodies against MVEV_TC123130_, which is consistent with previous studies using other JEV vaccines^106,107^, and is consistent with cross-reactivity in serology-based diagnostic assays^13,108^. There is also some evidence that JEV vaccination or infection provides partial cross-protection against MVEV and vice versa^109–111^.

Although one limitation of this study may be that JEV_NSW/22_ was isolated from a pig, there are only 4 amino acid differences between this isolate and a JEV G4 sequence from a human brain in the Tiwi Islands (Northern Territory, Australia) in 2021 (Genbank accession OM867669^14^). The differences are; envelope-238 F vs. L, NS2A-71 I vs. T, NS2B-59 E vs. G, NS3-436 E vs. G. In addition, JEV_Nakayama_ was passaged in suckling mouse brains, which may contribute to the increased virulence in C57BL/6J mice, although the adaptive mutations acquired during passaging, if any, are currently unknown. Furthermore, JEV_FU_ has not been passaged in mice, but was still more lethal than JEV_Nakayama_ in *Irf7*^*−/−*^ mice. The use of C57BL/6J mice that lack a functional nicotinamide nucleotide transhydrogenase *(Nnt)* may be another issue, as background and *Nnt* are able to affect viral immunopathogenesis^31,63^. However, we found that neither *Nnt* nor a C57BL/6N genetic background significantly impacted JEV replication or immunopathology (Supplementary Fig. 16).

In conclusion, we show that JEV_NSW/22_ has reduced virulence in mice but retains the capacity for rare lethal neuroinvasion, consistent with reported human fatalities in the 2022 Australian outbreak.

## Supplementary information

Supplementary Information

Supplementary Table 1

Supplementary Table 2

Supplementary Table 3

## Data Availability

All data are provided in the manuscript and accompanying supplementary files.
