# Supplementary material for: Characterisation of a Japanese Encephalitis virus genotype 4 isolate from the 2022 Australian outbreak
Source: Npj Viruses. 2024 May 10;2:15. doi: 10.1038/s44298-024-00025-5 (PMC11721158; doi:10.1038/s44298-024-00025-5)
Supplement: Supplementary file 1 — Supplementary Information [file 44298_2024_25_MOESM1_ESM.pdf]

A

Virus name	GenBank accession	JEV Genotype	Isolation year	Isolation source	Passage history
$JEV_{Nakayama}$	EF571853	3	1935	Human cerebrospinal fluid	>11 x suckling mouse brain
JEV_{FU}	AF217620	2	1995	Human serum	C6/36 (unknown number)
$JEV_{NSW2022}$	OP904182	4	2022	Stillborn piglet brain	1 x C6/36<br>1 x BHK-21
$MVEV_{TC123130}$	JN119814	N/A	1974	Human brain	Unknown
YFV 17D	MT107250	N/A	1927 (Original “Asibi” parent isolate)	Human serum (Original “Asibi” parent isolate)	53 x monkeys<br>18 x embryonic mouse tissue<br>50 x minced whole chicken embryo<br>152 x nervous tissue deprived chick embryo
JEV_{prME} component in Imojev (SA_{14}-14-2 strain)	MK585066	3	1954 (Original SA-14 parent isolate)	*Culex pipiens* mosquito larvae (Original SA-14 parent isolate)	100 x primary hamster kidney cells<br>22 x suckling mice

B

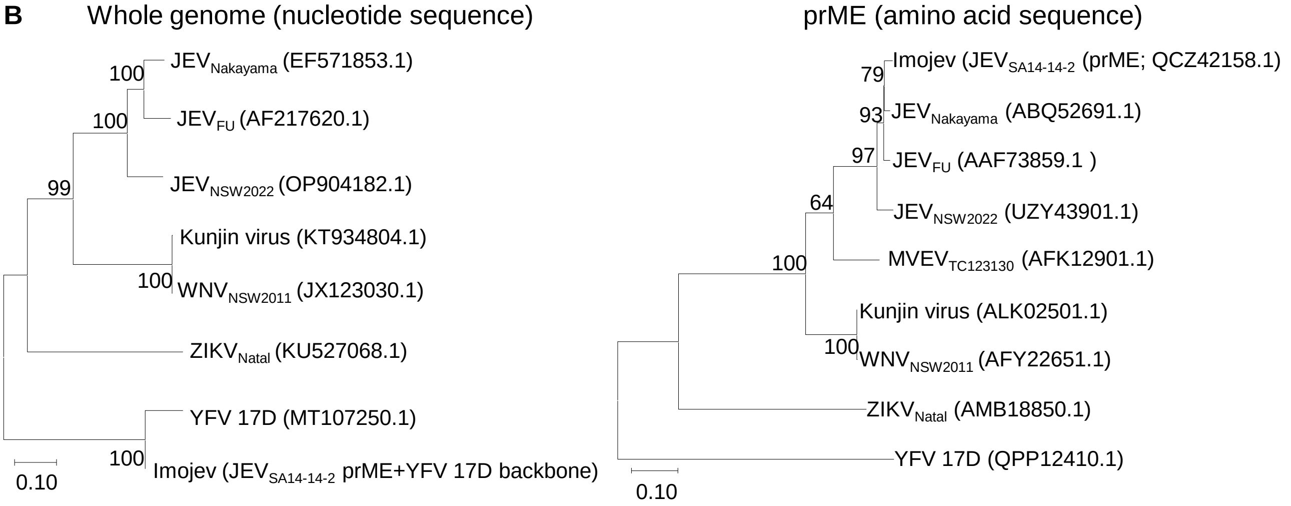

Supplementary Figure 1. (A) Summary of virus isolates used in the study. (B) Phylogenetic tree for whole genome nucleotide sequence (left) and prME amino acid sequence (right). Phylogenetic trees were constructed after nucleotide or amino acid sequence alignment using MEGA-X (Molecular Evolutionary Genetics Analysis 10, Penn State University, State College, PA, USA) and the ClustalW plugin with default parameters. The phylogenetic tree was constructed using the Maximum Likelihood method and the General Time Reversible model (nucleotide sequence) or JTT matrix-based model (amino acid sequence). Whole genome sequence is not available for $MVEV_{TC123130}$. Whole genome sequence for Imojev was constructed by combining the prME sequence from $JEV_{SA14-14-2}$ with the remainder of the genome from YFV 17D.

A

Activity

Dpi	JEV Nakayama Cg1 #2	Cg1 #6	Cg1 #2	Cg1 #3	Cg1 #6	Cg1 #4	JEV FU Cg2 #1	Cg3 #3	MVEV Cg1 #3	Cg4 #1
**0**	0	0	0	0	0	0	0	0	0	0
**1**	0	0	0	0	0	0	0	0	0	0
**2**	0	0	0	0	0	0	0	0	0	0
**3**	0	0	0	0	0	0	0	0	0	0
**4**	0	0	0	0	0	0	0	0	0	0
**5**	0	0	0	0	0	0	0	0	0	0
**6**	0	0	0	0	0	0	0	0	0	0
**7**	0	0	0	0	0	0	0	0	0	0
**8**	0	0	0	0	0	0	1	0	0	0
**9**	1	0	1	1	0	0		0	1	1
**10**		0	2	2	0	0		1		2
**11**		1			0	0		1		
**12**					2	0				
**13**						1				
**14**						2				

Weight loss score

Dpi	JEV Nakayama Cg1 #2	Cg1 #6	Cg1 #2	Cg1 #3	Cg1 #6	Cg1 #4	JEV FU Cg2 #1	Cg3 #3	MVEV Cg1 #3	Cg4 #1
**0**	0	0	0	0	0	0	0	0	0	0
**1**	0	0	0	0	0	0	0	0	0	0
**2**	0	0	0	0	0	0	0	0	0	0
**3**	0	0	0	0	0	0	0	0	0	0
**4**	0	0	0	0	0	0	0	0	0	0
**5**	0	0	0	0	0	0	0	0	0	0
**6**	0	0	0	0	0	0	0	1	0	0
**7**	0	0	0	0	0	0	2	2	0	0
**8**	1	0	0	0	0	0	3	3	2	0
**9**	3	0	1	1	0	0			3	1
**10**		1	3	3	0	0				3
**11**		3			1	0				
**12**					3	0				
**13**						2				
**14**						3				

Posture

Dpi	JEV Nakayama Cg1 #2	Cg1 #6	Cg1 #2	Cg1 #3	Cg1 #6	Cg1 #4	JEV FU Cg2 #1	Cg3 #3	MVEV Cg1 #3	Cg4 #1
**0**	0	0	0	0	0	0	0	0	0	0
**1**	0	0	0	0	0	0	0	0	0	0
**2**	0	0	0	0	0	0	0	0	0	0
**3**	0	0	0	0	0	0	0	0	0	0
**4**	0	0	0	0	0	0	0	0	0	0
**5**	0	0	0	0	0	0	0	0	0	0
**6**	0	0	0	0	0	0	0	0	0	0
**7**	0	0	0	0	0	0	0	0	0	0
**8**	0	0	0	0	0	0	1	0	0	0
**9**	1	0	1	1	0	0		0	1	1
**10**		0	1	1	0	0		1		1
**11**		1			0	0		1		
**12**					1	0				
**13**						1				
**14**						2				

Fur ruffling

Dpi	JEV Nakayama Cg1 #2	Cg1 #6	Cg1 #2	Cg1 #3	Cg1 #6	Cg1 #4	JEV FU Cg2 #1	Cg3 #3	MVEV Cg1 #3	Cg4 #1
**0**	0	0	0	0	0	0	0	0	0	0
**1**	0	0	0	0	0	0	0	0	0	0
**2**	0	0	0	0	0	0	0	0	0	0
**3**	0	0	0	0	0	0	0	0	0	0
**4**	0	0	0	0	0	0	0	0	0	0
**5**	0	0	0	0	0	0	0	0	0	0
**6**	0	0	0	0	0	0	0	0	0	0
**7**	0	0	0	0	0	0	0	0	0	0
**8**	0	0	0	0	0	0	0	0	0	0
**9**	1	0	0	0	0	0		0	1	0
**10**		0	0	0	0	0		0		0
**11**		0			0	0		1		
**12**					0	0				
**13**						0				
**14**						3				

B

**Criteria**	**Score 0**	**1 (mild)**	**2 (moderate)**	**3 (severe)**
Joint swelling	Normal	Slight to moderate swelling	Moderate/pronounce swelling and/or erythema	Joint distortion or rigidity
Injection site reaction	No reaction	Mild scaring	Moderate ulceration	Severe ulceration
Posture	Normal	Hunching noted only at rest	Severe hunching, some impairment of normal movement	Wobbling, unable to maintain upright posture
Activity	Normal	Mild to moderately decreased	Stationary unless stimulated.	Moderate paralysis, 1 or more limbs
Fur ruffling (fever sign)	Normal	Mild to moderate ruffling	Severe ruffling	Shivering
Hind leg weakness	Normal	Mild hind limb weakness/very mild and/or occasional gait alterations	Moderate hind limb weakness and occasional dragging of hind legs	Severe hind limb weakness/repeated dragging of hind legs
Weight loss	<10 %	10-15%	15-20%	≥20%

Supplementary Figure 2. Disease scores for the ten C57BL/6J mice that were euthanized.
(A) For the mice shown in Fig. 1F and I that were euthanized, the disease scores are shown. (B) The mice were monitored daily using the score card. Any animal reaching a level of 3 in any single criteria were euthanized. If an animal reaches a grade of 2 in two or more criteria the animal will be euthanized.

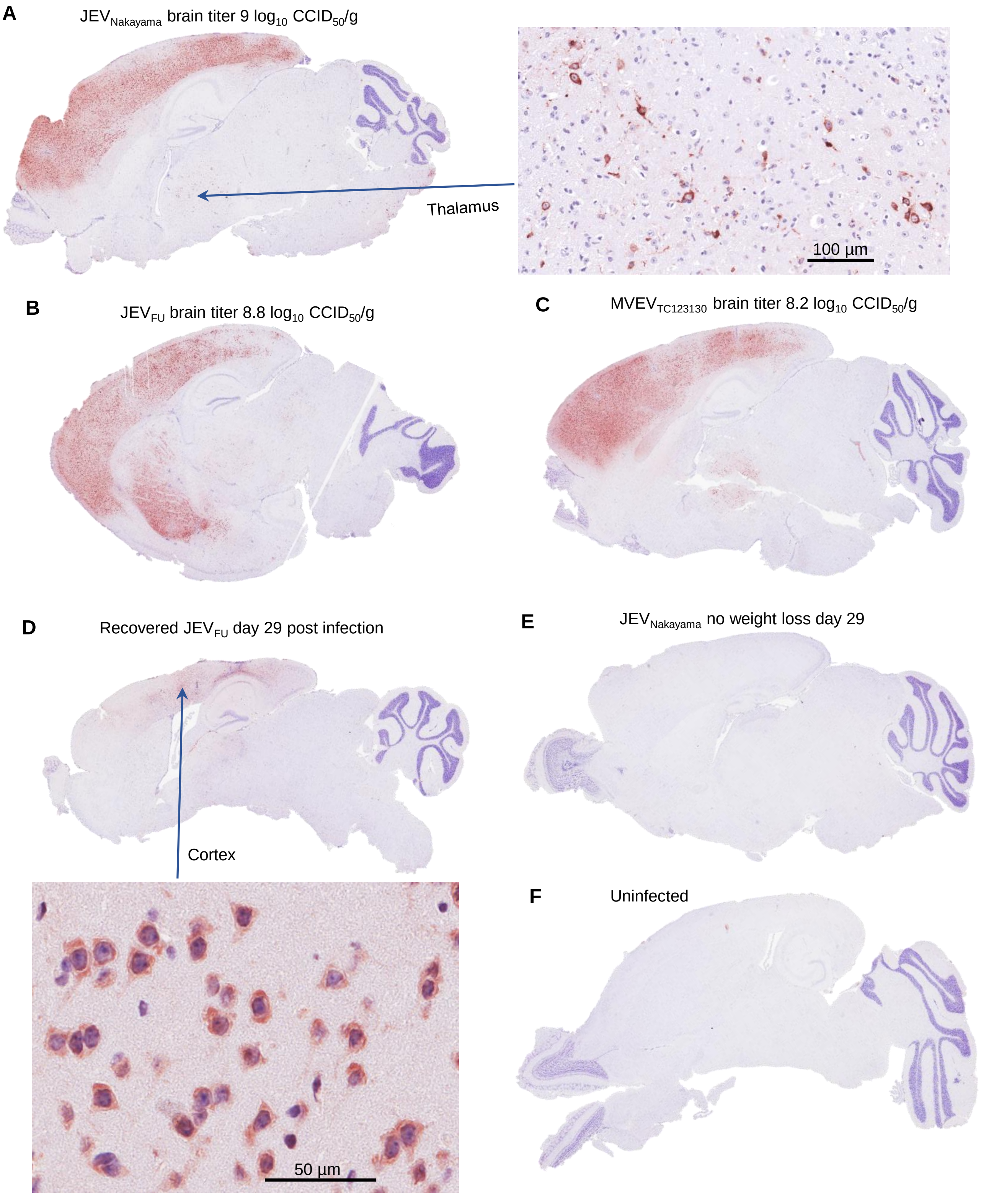

Supplementary Figure 3. Viral antigen (NS1) staining in C57BL/6J mouse brains. IHC using a pan-flavivirus NS1 monoclonal antibody (4G4). (A-C) The brains from the 3 other C57BL/6J mice that required euthanasia in Fig. 1 (the fourth is shown in Fig. 2). (D) JEV$_{FU}$ infected C57BL/6J mice that lost ~15% body weight then recovered (from Fig. 1E). (E) A representative image of a mouse brain where infection did not lead to significant weight loss. (F) Uninfected control.

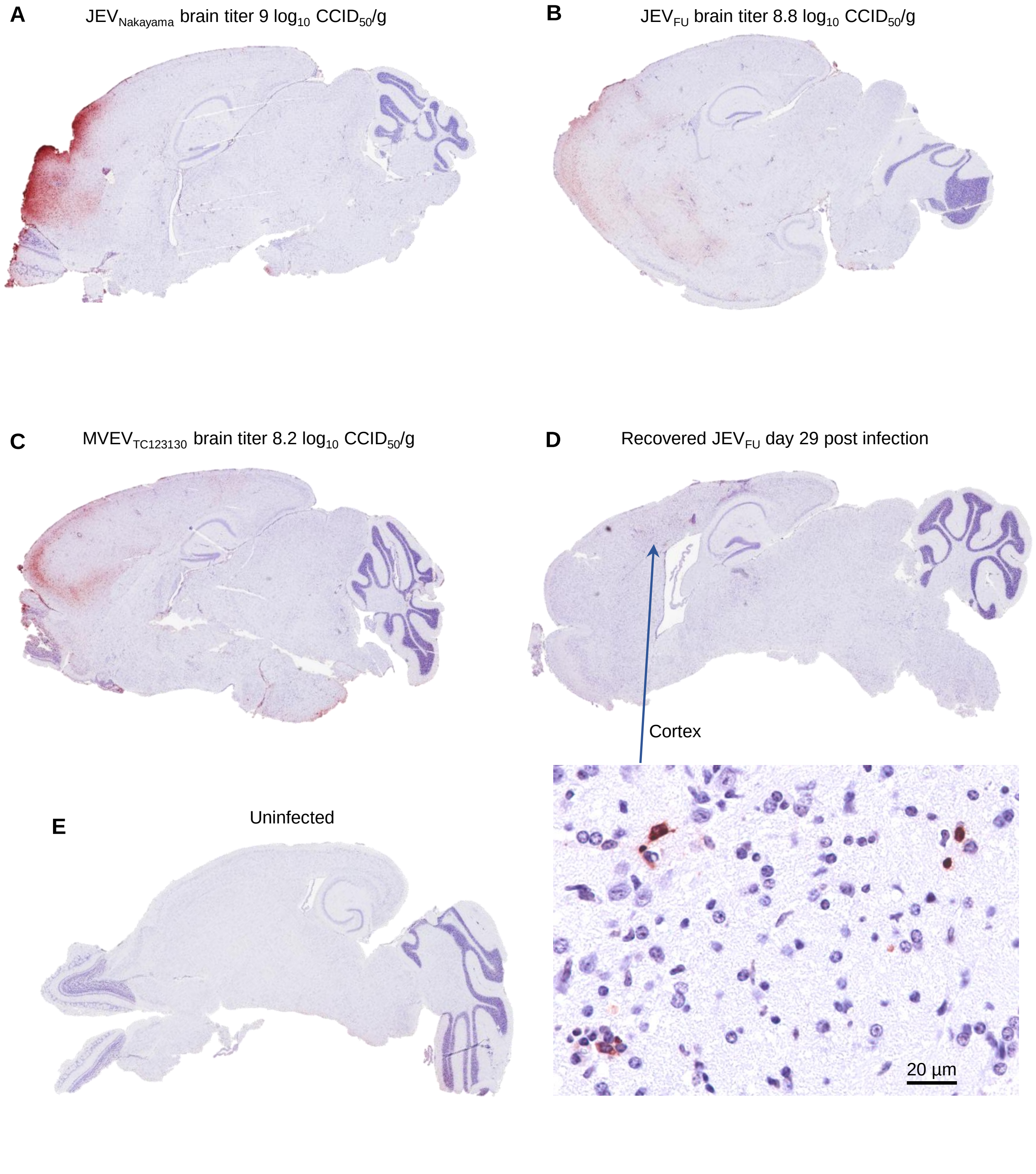

Supplementary Figure 4. ApopTag staining in C57Bl/6J mouse brains. Staining for apoptosis using Apoptag. (A-C) The brains from the 3 other C57BL/6J mice that required euthanasia in Figure 1. (D) The JEV$_{FU}$ infected C57BL/6J mice that lost ~15% body weight then recovered (see Figure 1E). (E) Uninfected control.

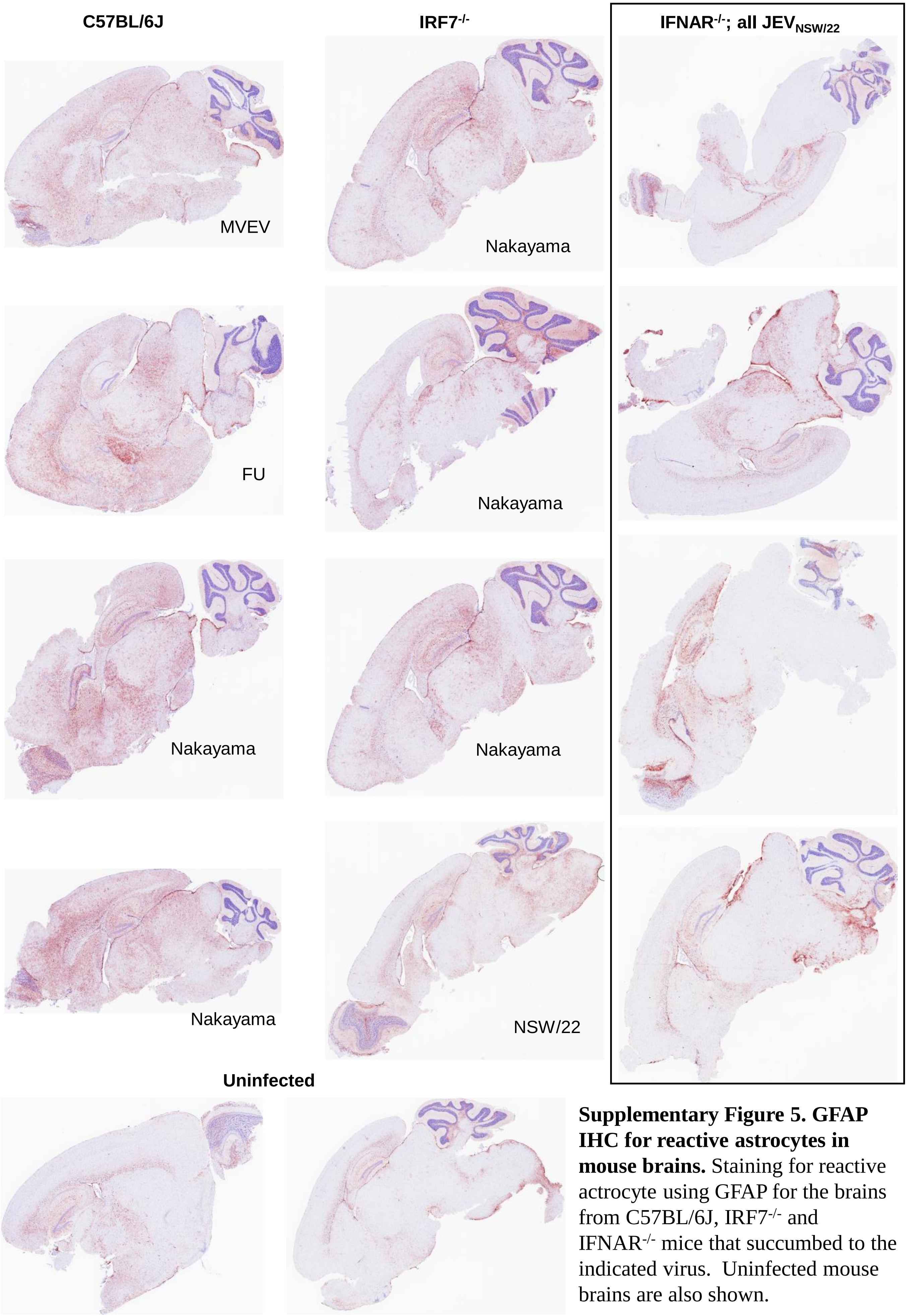

Supplementary Figure 5. GFAP IHC for reactive astrocytes in mouse brains. Staining for reactive actrocyte using GFAP for the brains from C57BL/6J, IRF7$^{-/-}$ and IFNAR$^{-/-}$ mice that succumbed to the indicated virus. Uninfected mouse brains are also shown.

Activity

	Nakayama								NSW/22	FU										MVEV
Dpi	Cg1#1	Cg1#2	Cg1#3	Cg5#1	Cg1#1	Cg3#1	Cg1#1	Cg1#2	Cg2#1	Cg19# 1	Cg19# 2	Cg19# 3	Cg19# 4	Cg11# 1	Cg12# 1	Cg14# 1	Cg14# 2	Cg3#2	Cg3#4	Cg4#4
**0**	0	0	0	0	0	0	0	0	0	0	0	0	0	0	0	0	0	0	0	0
**1**	0	0	0	0	0	0	0	0	0	0	0	0	0	0	0	0	0	0	0	0
**2**	0	0	0	0	0	0	0	0	0	0	0	0	0	0	0	0	0	0	0	0
**3**	0	0	0	0	0	0	0	0	0	0	0	0	0	0	0	0	0	0	0	0
**4**	0	0	0	0	0	0	0	0	0	0	0	0	0	0	0	0	0	0	0	0
**5**	0	0	0	0	0	0	0	0	0	0	0	0	0	0	0	0	0	0	0	0
**6**	0	0	0	0	0	0	0	0	0	0	3	0	2	1	0	0	0	0	0	0
**7**	0	1	0	1	0	0		2	0	2		2		1	0	2	0	2	2	0
**8**	0		2	1	0	0			1					2	0		2			0
**9**	0				2	0									1					0
**10**	0														1					0
**11**	1																			0
**12**	2																			0

Posture

	Nakayama								NSW/22	FU										MVEV
Dpi	Cg1#1	Cg1#2	Cg1#3	Cg5#1	Cg1#1	Cg3#1	Cg1#1	Cg1#2	Cg2#1	Cg19# 1	Cg19# 2	Cg19# 3	Cg19# 4	Cg11# 1	Cg12# 1	Cg14# 1	Cg14# 2	Cg3#2	Cg3#4	Cg4#4
**0**	0	0	0	0	0	0	0	0	0	0	0	0	0	0	0	0	0	0	0	0
**1**	0	0	0	0	0	0	0	0	0	0	0	0	0	0	0	0	0	0	0	0
**2**	0	0	0	0	0	0	0	0	0	0	0	0	0	0	0	0	0	0	0	0
**3**	0	0	0	0	0	0	0	0	0	0	0	0	0	0	0	0	0	0	0	0
**4**	0	0	0	0	0	0	0	0	0	0	0	0	0	0	0	0	0	0	0	0
**5**	0	0	0	0	0	0	0	0	0	0	0	0	0	0	0	0	0	0	0	0
**6**	0	0	0	0	0	0	0	0	0	0	2	0	2	1	0	0	0	0	0	0
**7**	0	2	0	1	0	0		1	0	1		1		1	0	1	0	2	2	0
**8**	0		2	1	0	0			1					2	0		2			1
**9**	0				2	1									1					1
**10**	0														2					0
**11**	1																			1
**12**	2																			1

Weight loss score

	Nakayama								NSW/22	FU										MVEV
Dpi	Cg1# 1	Cg1# 2	Cg1# 3	Cg5# 1	Cg1# 1	Cg3# 1	Cg1# 1	Cg1# 2	Cg2#1	Cg19# 1	Cg19# 2	Cg19# 3	Cg19# 4	Cg11# 1	Cg12# 1	Cg14# 1	Cg14# 2	Cg3# 2	Cg3# 4	Cg4# 4
**0**	0	0	0	0	0	0	0	0	0	0	0	0	0	0	0	0	0	0	0	0
**1**	0	0	0	0	0	0	0	0	0	0	0	0	0	0	0	0	0	0	0	0
**2**	0	0	0	0	0	0	0	0	0	0	0	0	0	0	0	0	0	0	0	0
**3**	0	0	0	0	0	0	1	0	0	0	0	0	0	0	0	0	0	0	0	0
**4**	0	0	0	0	0	0	1	0	0	0	0	0	0	0	0	0	0	0	0	0
**5**	0	0	0	0	0	0	2	0	0	0	0	0	0	0	0	0	0	0	0	0
**6**	0	0	0	1	0	0	3	0	0	0	1	0	1	2	0	0	0	1	0	0
**7**	0	2	0	2	0	1		2	0	3		2		2	1	2	0	3	2	1
**8**	0		1	3	1	2			3					3	1		3			1
**9**	0				3	3									2					1
**10**	0														3					2
**11**	2																			2
**12**	3																			3

Fur ruffling

	Nakayama								NSW/22	FU										MVEV
Dpi	Cg1# 1	Cg1# 2	Cg1# 3	Cg5# 1	Cg1# 1	Cg3# 1	Cg1# 1	Cg1# 2	Cg2#1	Cg19# 1	Cg19# 2	Cg19# 3	Cg19# 4	Cg11# 1	Cg12# 1	Cg14# 1	Cg14# 2	Cg3# 2	Cg3# 4	Cg4# 4
**0**	0	0	0	0	0	0	0	0	0	0	0	0	0	0	0	0	0	0	0	0
**1**	0	0	0	0	0	0	0	0	0	0	0	0	0	0	0	0	0	0	0	0
**2**	0	0	0	0	0	0	0	0	0	0	0	0	0	0	0	0	0	0	0	0
**3**	0	0	0	0	0	0	0	0	0	0	0	0	0	0	0	0	0	0	0	0
**4**	0	0	0	0	0	0	0	0	0	0	0	0	0	0	0	0	0	0	0	0
**5**	0	0	0	0	0	0	0	0	0	0	0	0	0	0	0	0	0	0	0	0
**6**	0	0	0	0	0	0	0	0	0	0	0	0	1	1	0	0	0	0	0	0
**7**	0	2	0	1	0	0		0	0	0		0		1	0	0	0	0	0	0
**8**	0		1	1	1	0			1					1	0		1			0
**9**	0				3	1									1					0
**10**	0														2					0
**11**	0																			0
**12**	0																			0

Supplementary Figure 6. Disease scores for IRF7-/- mice that were euthanized. Mice described in Figure 3C, D. Scoring system shown in Supplementary Figure 2B.

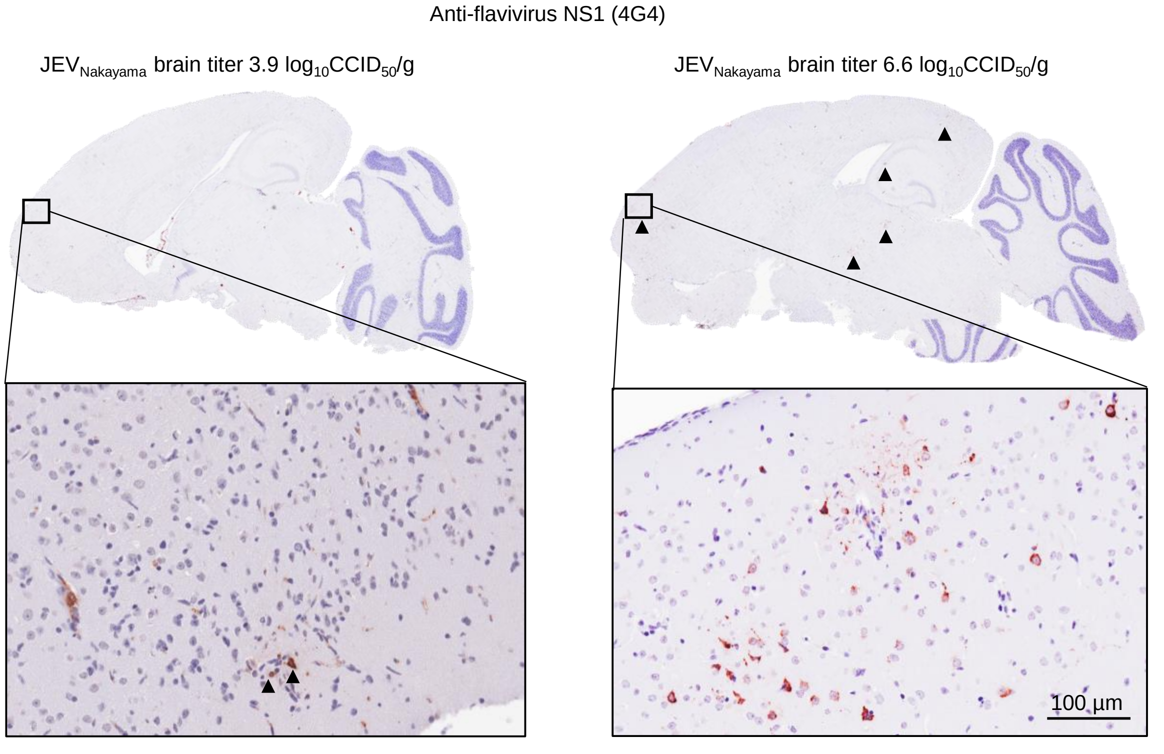

Supplementary Figure 7. Viral antigen staining in IRF7$^{-/-}$ mouse brains. IHC using a pan-flavivirus NS1 monoclonal antibody (4G4) for the brains from 2 other IRF7$^{-/-}$ mice that required euthanasia (Figure 3). Low levels of staining of neurons were found in the JEV$_{Nakayama}$ infected mice that had a low virus titer in the brain (3.9 $log_{10}CCID_{50}$/g) (left). Black arrowheads show small patches of staining in the cortex, hippocampus and thalamus (right).

A

Activity	FU					Nakayama				NSW/22				$MVEV_{TC123130}$			
	Cg4#	Cg4#	Cg4#	Cg4#	Cg1#	Cg2#	Cg5#	Cg5#	Cg5#	Cg3#	Cg3#	Cg3#	Cg3#	Cg3#	Cg3#	Cg3#	Cg3#
**Dpi**	1	2	3	4	1	1	1	2	3	1	2	3	4	1	2	3	4
**0**	0	0	0	0	0	0	0	0	0	0	0	0	0	0	0	0	0
**1**	0	0	0	0	1	1	0	0	0	0	0	0	0	0	0	0	0
**2**	1	1	1	1	2	1	0	0	0	1	1	1	1	0	0	0	0
**3**	2	2	2	2		2	2	2	2	2	2	2	2	1	1	2	1

Posture	FU					Nakayama				NSW/22				$MVEV_{TC123130}$			
	Cg4#	Cg4#	Cg4#	Cg4#	Cg1#	Cg2#	Cg5#	Cg5#	Cg5#	Cg3#	Cg3#	Cg3#	Cg3#	Cg1#	Cg1#	Cg1#	Cg1#
**Dpi**	1	2	3	4	1	1	1	2	3	1	2	3	4	1	2	3	4
**0**	0	0	0	0	0	0	0	0	0	0	0	0	0	0	0	0	0
**1**	0	0	0	0	0	0	0	0	0	0	0	0	0	0	0	0	0
**2**	1	1	1	1	1	1	1	1	0	0	0	0	0	1	0	0	0
**3**	2	2	2	2		2	2	2	2	2	2	2	2	2	2	1	2

Fur ruffling	FU					Nakayama				NSW/22				$MVEV_{TC123130}$			
	Cg4#	Cg4#	Cg4#	Cg4#	Cg1#	Cg2#	Cg5#	Cg5#	Cg5#	Cg3#	Cg3#	Cg3#	Cg3#	Cg1#	Cg1#	Cg1#	Cg1#
**Dpi**	1	2	3	4	1	1	1	2	3	1	2	3	4	1	2	3	4
**0**	0	0	0	0	0	0	0	0	0	0	0	0	0	0	0	0	0
**1**	0	0	0	0	0	0	0	0	0	0	0	0	0	0	0	0	0
**2**	1	1	1	1	0	0	0	0	0	0	0	0	0	0	0	0	0
**3**	1	1	1	1		0	1	1	1	0	0	0	0	1	1	2	1

Weight loss score	FU					Nakayama				NSW/22				$MVEV_{TC123130}$			
	Cg4#	Cg4#	Cg4#	Cg4#	Cg1#	Cg2#	Cg5#	Cg5#	Cg5#	Cg3#	Cg3#	Cg3#	Cg3#	Cg1#	Cg1#	Cg1#	Cg1#
**Dpi**	1	2	3	4	1	1	1	2	3	1	2	3	4	1	2	3	4
**0**	0	0	0	0	0	0	0	0	0	0	0	0	0	0	0	0	0
**1**	1	1	0	0	0	0	0	0	0	0	0	0	0	0	0	0	0
**2**	2	1	2	2	2	1	1	1	1	0	0	0	0	0	0	0	0
**3**	2	1	2	2		3	2	2	2	2	1	2	1	2	2	2	2

B

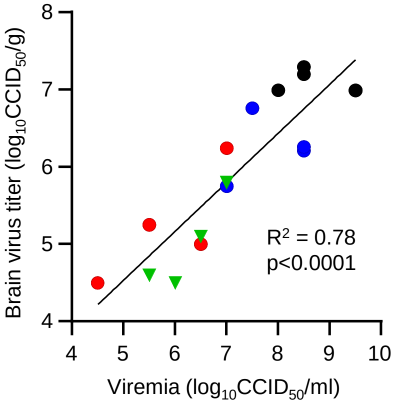

C

$JEV_{NSW/22}$ brain $IFNAR^{-/-}$ mouse, 3 dpi, titer 6.6 log_{10} $CCID_{50}$/g

Anti-flavivirus NS1 | ApopTag

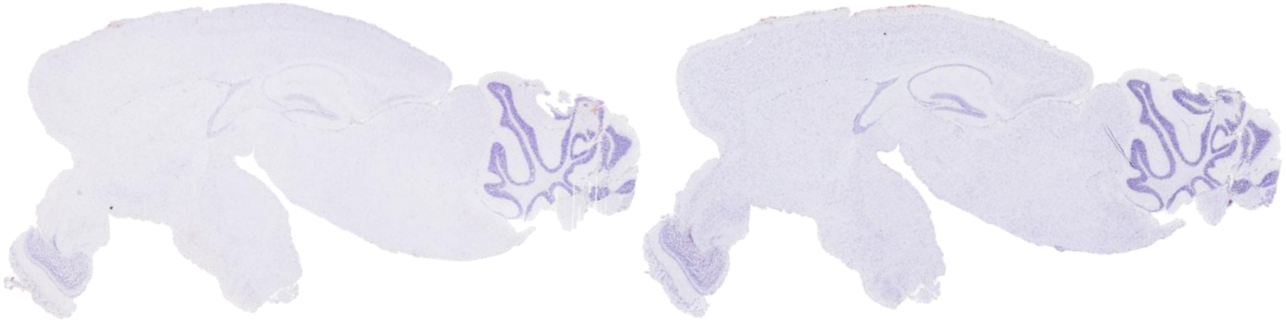

Supplementary Figure 8. JEV and MVEV infection of $IFNAR^{-/-}$ mice. (A) Disease scores as per Supplementary Figure 2B for mice shown in Figure 3. (B) Pearson correlation between brain titer (y-axis) from Figure 3E and viremia (x-axis) from Figure 3A. (B) IHC for flavivirus NS1 (left) or ApopTag (right) showing no detectable staining of brain cells. Images are representative of all $IFNAR^{-/-}$ brains and JEV isolates.

A Acute (euthanized due to weight loss/disease scores)

Mouse	C57BL/6J			*Irf7*$^{-/-}$			*Ifnar*$^{-/-}$		
**JEV isolate**	NSW	Nakayama	FU	NSW	Nakayama	FU	NSW	Nakayama	FU
**Euthanasia**		9-14 dpi	8-12 dpi	8 dpi	7-12 dpi	6-10 dpi	3 dpi	3 dpi	3 dpi
Neuronal degeneration /vacuolation	**N/A**	**6/6**	**2/2**	**1/1**	**3/8**	**7/10**	**0/4**	**0/3**	**0/4**
Perivascular cuffing	**N/A**	**6/6**	**2/2**	**1/1**	**8/8**	**10/10**	**0/4**	**0/3**	**0/4**
Hemorrhage	**N/A**	**5/6**	**1/2**	**0/1**	**5/8**	**6/10**	**3/4**	**2/3**	**2/4**
Leukocyte infiltrates	**N/A**	**5/6**	**2/2**	**1/1**	**1/8**	**3/10**	**0/4**	**0/3**	**0/4**
Microgliosis	**N/A**	**6/6**	**2/2**	**1/1**	**5/8**	**7/10**	**0/4**	**0/3**	**0/4**
Meningitis	**N/A**	**6/6**	**2/2**	**1/1**	**6/8**	**8/10**	**0/4**	**0/3**	**0/3**

B

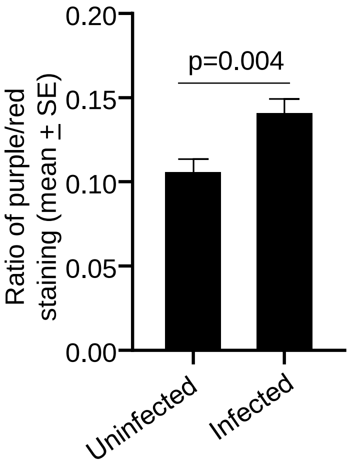

C Chronic (euthanized after recovery from acute phase)

Mouse	C57BL/6J			*Irf7*$^{-/-}$		
**JEV isolate**	NSW	Nakayama	FU	NSW	Nakayama	FU
**Euthanasia**	32 dpi	32 dpi	32 dpi	19-21 dpi	19-21 dpi	19-21 dpi
Neuronal degeneration /vacuolation	**0/6**	**0/4**	**0/5**	**0/13**	**0/6**	**0/4**
Perivascular cuffing	**1/6**	**0/4**	**1/5**	**2/13**	**5/6**	**1/4**
Hemorrhage	**3/6**	**2/4**	**1/5**	**5/13**	**5/6**	**1/4**
Leukocyte infiltrates	**0/6**	**0/4**	**1/5**	**0/13**	**0/6**	**0/4**
Microgliosis	**0/6**	**0/4**	**2/5**	**2/13**	**5/6**	**2/4**
Meningitis	**0/6**	**0/4**	**0/5**	**1/13**	**0/6**	**1/4**

Supplementary Figure 9. Lesion presence in H&E brain sections. A) Scoring for lesions described in Figure 6 for mice with acute disease. H&E scoring of 0 indicates no overt presence of these lesions. N/A – not available. B) Ratio of nuclear (blue/dark purple) to non-nuclear (red) staining of H&E stained brain sections (a measure of leukocyte infiltration). Data is the mean and standard error for n=30 infected and n=14 uninfected mouse brains from C57BL/6J and *Irf7*$^{-/-}$ mice that succumbed to JEV or MVEV infection (Fig. 1F, 1I and 3D). Statistics by t-test. C) As for 'A' but for mice that survived infection.

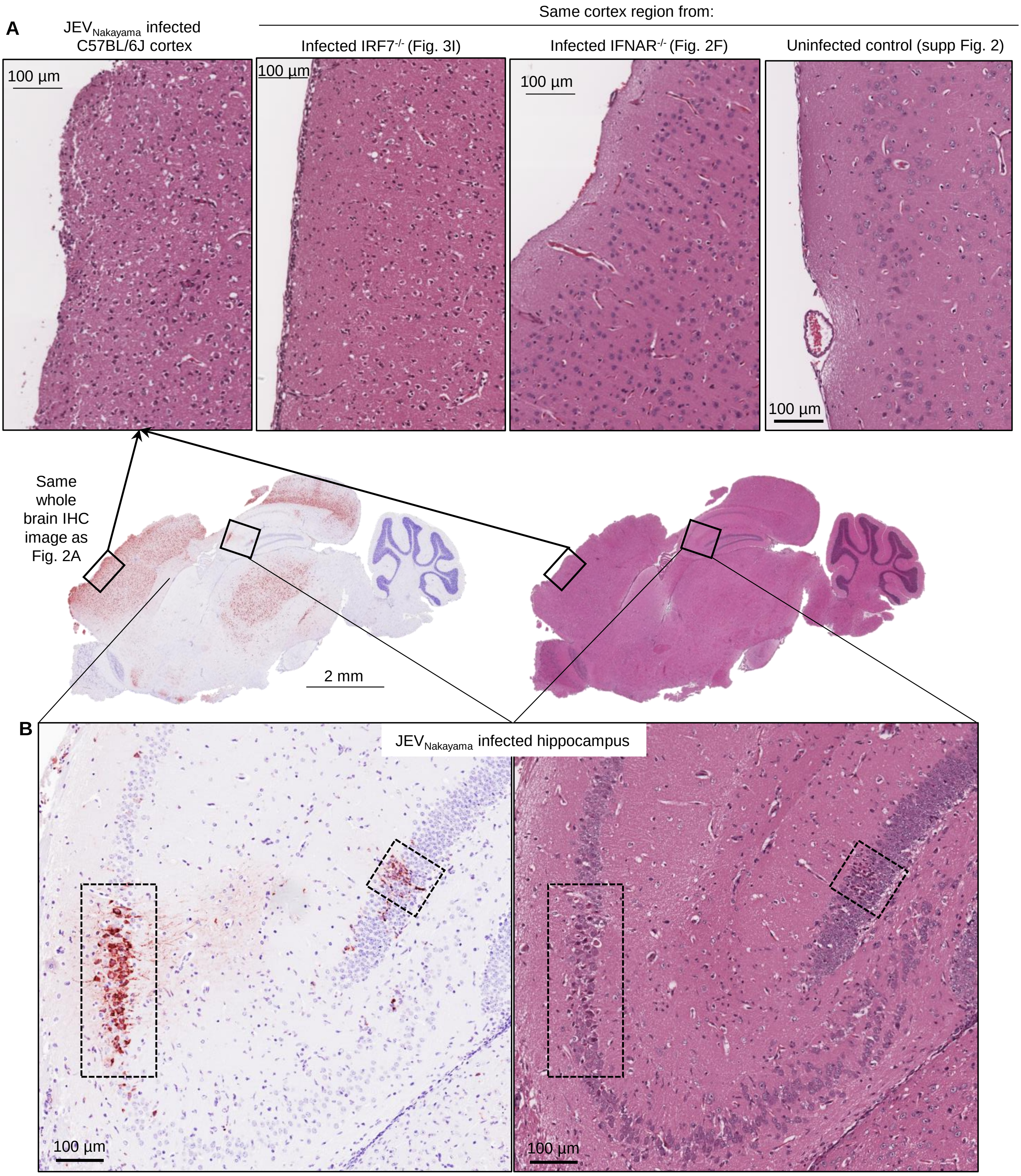

Supplementary Figure 10. Histopathological lesions overlapped with areas of virus infection. (A) Cortex regions from JEV infected C57BL/6J, IRF7$^{-/-}$, IFNAR$^{-/-}$ mice or uninfected mice. The cortex regions from JEV infected mice were heavily infected, with most neurons stained positive for viral antigen. H&E detectable signs of neuron degeneration/vacuolation were concentrated in regions with high staining for viral antigen. (B) Viral antigen staining (left) in hippocampus overlapped with histological signs of neuron degeneration/vacuolation (right) (see Figure 6).

A **IRF7$^{-/-}$ and C57BL/6J mice (n=27) that were euthanized due to ethically defined disease end points**

	Neuron degeneration/ vacuolation	Perivascular cuffing	Hemorrhage	Leukocyte infiltrates	Microgliosis
Cerebral cortex	17/27	20/27	8/27	8/27	8/27
Hypothalamus	0/27	827	3/27	2/27	7/27
Thalamus	0/27	16/27	2/27	2/27	9/27
Hippocampus	0/27	10/27	0/27	1/27	8/27
Caudate putamen	1/27	16/27	1/27	5/27	7/27
Basal forebrain	0/27	12/27	2/27	0/27	0/27
Midbrain	0/27	7/27	1/27	0/27	5/27
Pons	0/27	6/27	6/27	1/27	1/27
Medulla	0/27	4/27	0/27	0/27	0/27
Cerebellum	0/27	6/27	1/27	0/27	0/27
Anterior olfactory nucleus	0/27	3/27	0/27	0/27	0/27
Ventral striatum	0/27	7/27	0/27	0/27	0/27

B **IRF7$^{-/-}$ and C57BL/6J mice (n=38) that survived infection (taken on day 19-21 and day 32, respectively)**

	Neuron degeneration/ vacuolation	Perivascular cuffing	Hemorrhage	Leukocyte infiltrates	Microgliosis
Cerebral cortex	0/38	4/38	7/38	1/38	5/38
Hypothalamus	0/38	0/38	2/38	0/38	0/38
Thalamus	0/38	2/38	1/38	0/38	4/38
Hippocampus	0/38	0/38	0/38	0/38	0/38
Caudate putamen	0/38	4/38	0/38	0/38	5/38
Basal forebrain	0/38	1/38	5/38	0/38	0/38
Midbrain	0/38	0/38	0/38	0/38	2/38
Pons	0/38	0/38	3/38	0/38	1/38
Medulla	0/38	0/38	1/38	0/38	0/38
Cerebellum	0/38	1/38	2/38	0/38	0/38
Anterior olfactory nucleus	0/38	0/38	1/38	0/38	0/38
Ventral striatum	0/38	0/38	2/38	0/38	1/38

Supplementary Figure 11. Histopathological lesions in specific brain regions for C57BL/6J and IRF7$^{-/-}$. Scoring only reflects presence or absence of lesions, and does not indicate severity of lesions. (A) Acute (B) Chronic.

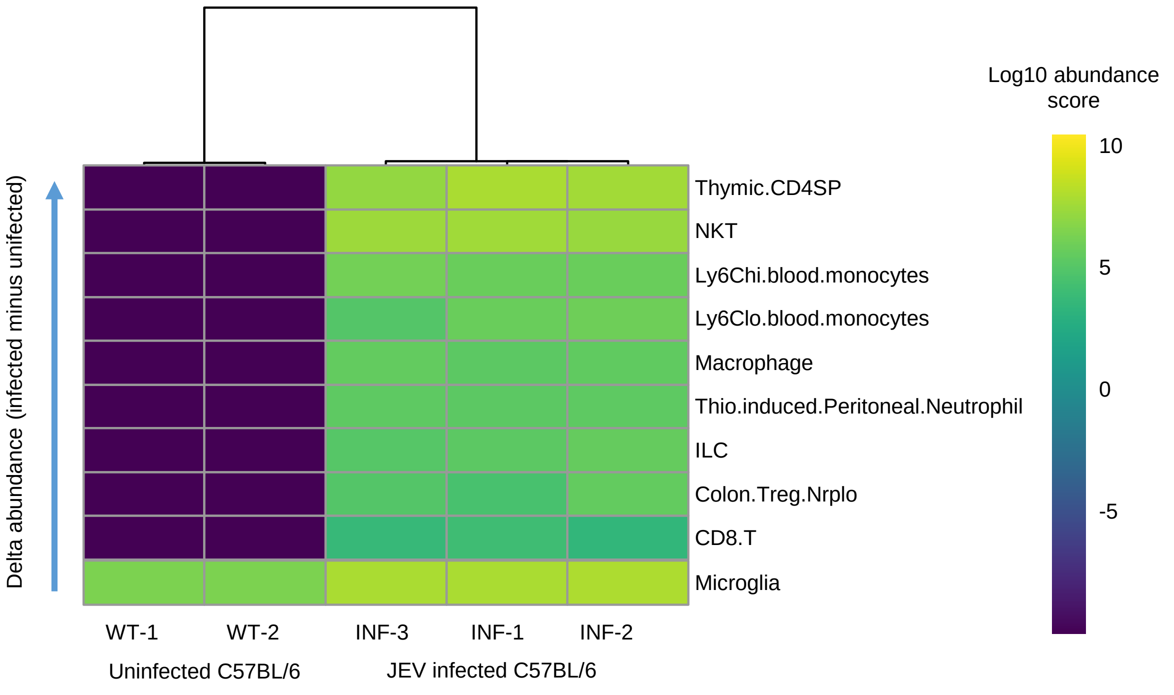

Supplementary Figure 12. RNA-Seq read counts from brains of C57BL/6 JEV-infected and mock-infected mice obtained from the Gene Expression Omnibus (GEO accession: GSE154002) were normalised for sequencing depth and composition using DESeq2. An estimation of cell type abundances was performed on normalised read counts with the SpatialDecon package in R, using an adult mouse immune cell gene expression reference (Yoshida *et al.* 2019. Cell 176; 897-912.e20). Log_{10} abundance scores are shown for cell types that were significantly different between infected and mock-infected groups (t-test, p-value < 0.05), with cell types ordered from largest to smallest difference in mean abundance score between groups.

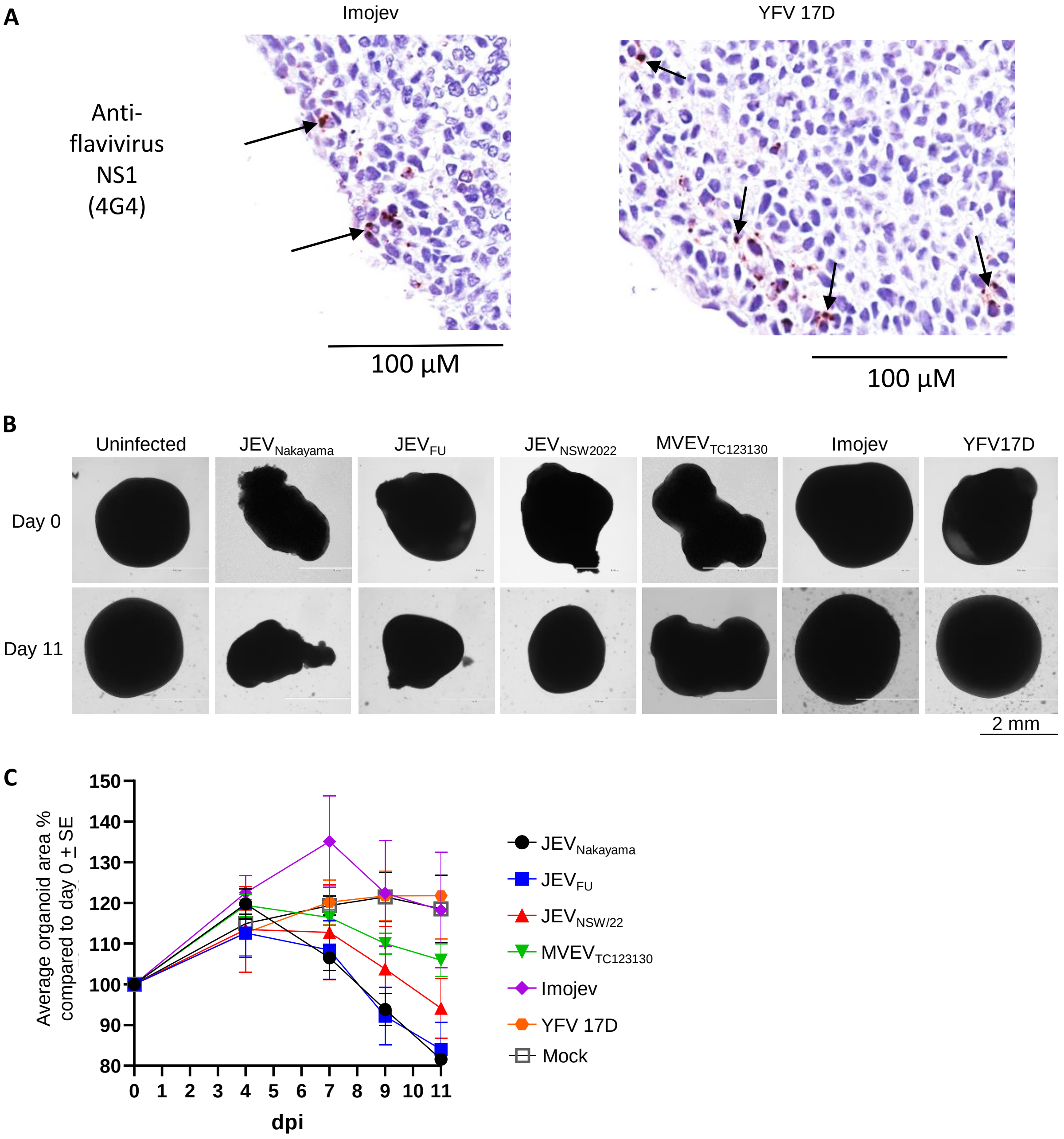

Supplementary Fig. 13. Human cortical brain organoids measurements and magnified virus staining. (A) Occasional cells staining positive for IHC for viral antigen in hBOs infected with Imojev or YFV 17D. (B) Light microscopy images of hBOs infected with $JEV_{Nakayama}$, JEV_{FU}, $JEV_{NSW2022}$, $MVEV_{TC123130}$, Imojev or YFV 17D at day 0 and day 11 post infection compared to uninfected hBOs. Scale bar is consistent across all images. (C) Change in hBO area on day 4, 7, 9 and 11 compared to day 0. n=8 for uninfected and $MVEV_{TC123130}$, n=4 for all others.

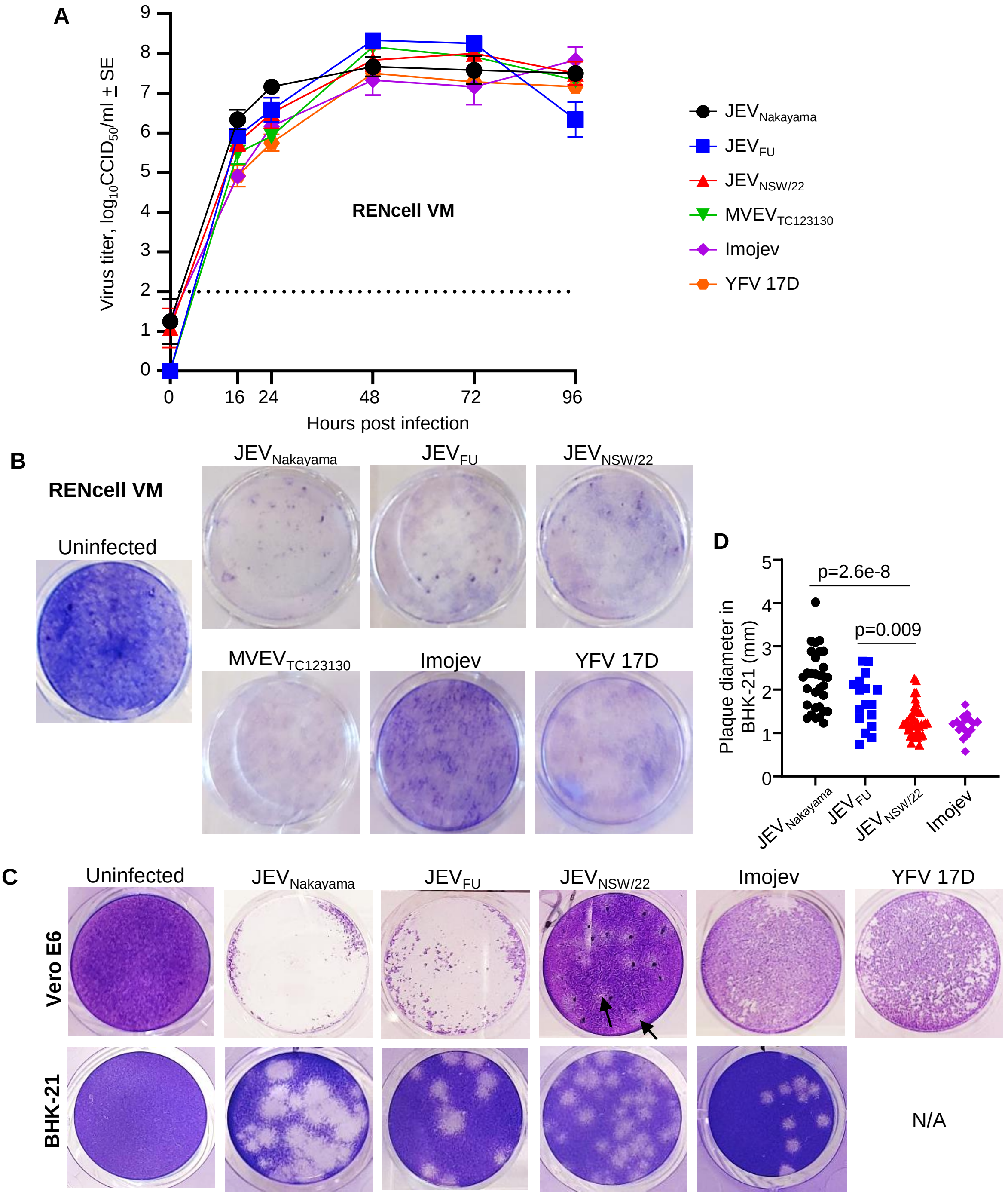

Supplementary Figure 14. Replication and CPE in RENcell VM neural progenitor and Vero E6 cell lines. (A) Viral growth kinetics in RENcell VM determined by $CCID_{50}$ assays of culture supernatant at the indicated times post infection. Data is the mean of 6 replicates per virus isolate across 2 independent experiments; (limit of detection is 2 $log_{10}CCID_{50}$/ml). (B) Images of crystal violet stained RENcell VM at 6 dpi (representative of n=3 per group). Less blue/violet stained cells indicates more viral CPE. (C) Vero or BHK-21 cells were seeded at $2.5x10^5$ cells per well in 24 well plates overnight at 37°C. Cells were infected at MOI=0.05 for 1 hr at 37°C before overlay media (0.375% w/v high viscosity carboxymethyl cellulose [CMC, Sigma-Aldrich]/RPMI 1640 substituted with 2% FCS) was added to each well. Plates were incubated for 5 days before the media was removed and monolayers were fixed and stained with 0.1% w/v crystal violet (Sigma-Aldrich) in formaldehyde (1% v/v) and methanol (1% v/v). Plates were washed in tap water and dried before images were taken. Images shown are representative of n=4 per group. D) Plaque diameter measurements for BHK-21 cells shown in ‘C’, measured using Image J.

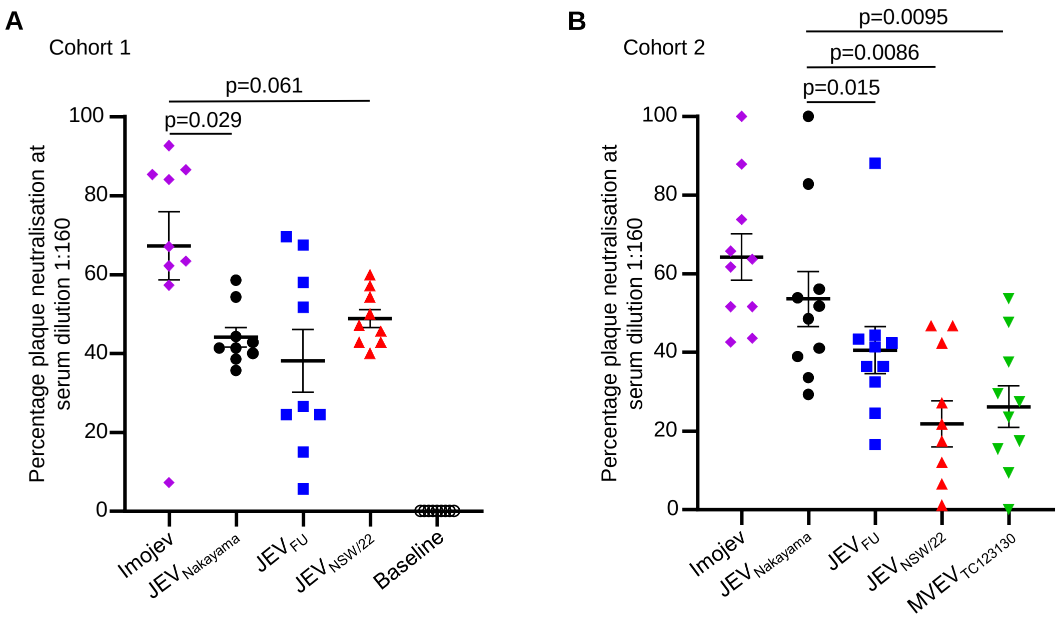

Supplementary Fig. 15. Percentage neutralisation of JEV and MVEV at serum dilution 1:160. (A) Human serum from cohort 1 (n=9) taken taken pre-vaccination (baseline) and day 28 post-Imojev vaccination was used in plaque reduction neutralisation assays against Imojev, $JEV_{Nakayama}$, $JEV_{NSW/22}$ and JEV_{FU}. (B) Human serum from cohort 2 (n=10) taken >2 months post-Imojev vaccination was used in plaque reduction neutralisation assays against Imojev, $JEV_{Nakayama}$, $JEV_{NSW/22}$ and JEV_{FU} and $MVEV_{TC123130}$. Individual data points in both A and B represent the mean percentage neutralisation at serum dilution 1:160 from duplicate wells in $PRNT_{50}$ assay. The mean of all individuals (n=9 from cohort 1 and n=10 from cohort 2) and standard error is shown. Statistics are by paired t-test.

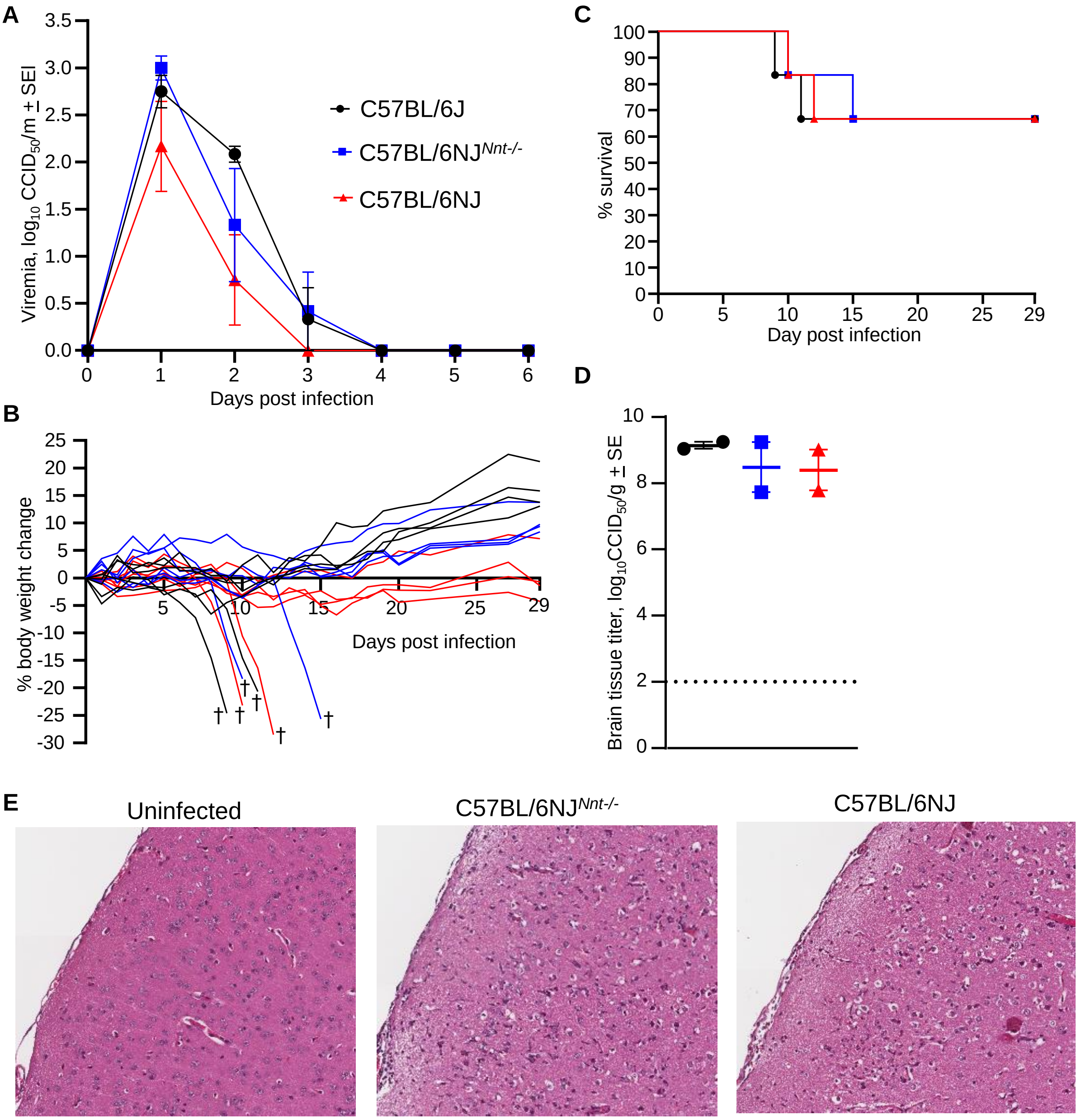

Supplementary Fig. 16. Neither mouse background nor *Nnt* significantly affected JEV neuropathogenesis. Female C57BL/6J (10-16 weeks old) (black circles), C57BL/6NJ$^{Nnt-/-}$ (blue squares), and C57BL/6NJ (red triangles) mice were infected s.c. with 5x10^5 $CCID_{50}$ $JEV_{Nakayama}$. (A) Average viremia as determined by $CCID_{50}$ assays; limit of detection for individual mice is 2 $log_{10}CCID_{50}$/ml. (B) Percent body weight change of individual mice compared to their weight 0 dpi. Six mice lost ≥20% body weight and required euthanasia (†). (C) Kaplan Myer plot showing percent survival (n=6 for each mouse strain). (D) Viral tissue titers in brains of the six euthanized mice (n=2 from each mouse strain). Tissue titers determined by $CCID_{50}$ assays (limit of detection ~2 $log_{10}CCID_{50}$/g). (E) Representative images of H&E stained sections of brains from mice that required euthanasia. No overt differences were identified between C57BL/6J, C57BL/6NJ$^{Nnt-/-}$, or C57BL/6NJ mice.
